# Southern African HIV Clinicians Society guidelines for harm reduction

**DOI:** 10.4102/sajhivmed.v21i1.1161

**Published:** 2020-12-17

**Authors:** Andrew Scheibe, Goodman Sibeko, Shaun Shelly, Theresa Rossouw, Vincent Zishiri, Willem D.F. Venter

**Affiliations:** 1 TB HIV Care, Cape Town, South Africa; 2 Department of Family Medicine, University of Pretoria, Pretoria, South Africa; 3 Department of Psychiatry and Mental Health, University of Cape Town, Cape Town, South Africa; 4 Department of Immunology, University of Pretoria, Pretoria, South Africa; 5 Ezintsha, Faculty of Health Sciences, University of the Witwatersrand, Johannesburg, South Africa

## Table of contents

**Executive summary**

1. Introduction
  1.1 Harm reduction
  1.2 Drugs and drug use
    1.2.1 Patterns of use
    1.2.2 Methods of drug use
    1.2.3 Drug classifications and common drugs
    1.2.4 Epidemiology of drug use in southern Africa
    1.2.5 Drug-related harms
    1.2.6 Reasons for drug use
    1.2.7 The relevance of harm reduction
  1.3 Stigma, discrimination and human rights
2. Evidence-based interventions
  2.1 A guiding framework
  2.2 Assessing a person’s needs
    2.2.1 Screening and brief intervention for common mental disorders and harmful substance use
      2.2.1.1 Screening
      2.2.1.2 Brief interventions
      2.2.1.3 Referral for treatment
  2.3 HIV prevention
    2.3.1 Condoms and lubricant
    2.3.2 Pre- and post-exposure prophylaxis
    2.3.3 Voluntary medical male circumcision
  2.4 Harm reduction interventions
    2.4.1 Needle-and-syringe services
    2.4.2 Opioid substitution therapy
    2.4.3 Overdose services
    2.4.4 Emerging and ancillary interventions
      2.4.4.1 Harm reduction for people who use stimulants
      2.4.4.2 Drug consumption rooms
      2.4.4.3 Drug-checking services
  2.5 HIV testing, treatment and care
    2.5.1 HIV testing and counselling
    2.5.2 Antiretroviral therapy
    2.5.3 Prevention of mother-to-child transmission of HIV
  2.6 Prevention and management of coinfections and comorbidities
    2.6.1 Tuberculosis services
    2.6.2 Viral hepatitis services
    2.6.3 Mental health services
    2.6.4 Sexual and reproductive health services
  2.7 Critical enablers
    2.7.1 Supportive law and policy
    2.7.2 Countering stigma and discrimination
    2.7.3 Enabling community empowerment
    2.7.4 Acting against violence
3. Special considerations
  3.1 Young people who use drugs
  3.2 Women who use drugs
  3.3 Substance use in the context of sexual encounters
  3.4 Prison settings
4. Recommendations
5. Acknowledgements
  Glossary
  References

Appendix 1: Common myths about drugs use
Appendix 2: Assessing patients during first and subsequent encounters
Appendix 3: Guidelines for opioid substitution therapy
Appendix 4: Psychosocial and mental health interventions

## Executive summary

We support public-health-focused interventions, as opposed to recovery-focused interventions. We support the decriminalisation of drug use as much as we oppose the criminalisation of sex work, mandatory HIV disclosure and policing of sexual preferences.

In South Africa, despite existing policy that embraces drug harm reduction, population- and individual-level interventions have focused largely on the singular goal of abstinence. This greatly impacts the human rights of people who use drugs and their communities. The failure of countries to implement comprehensive harm reduction measures violates their obligations in international human rights law and public health.

These guidelines were developed to provide information for healthcare workers working in the field of HIV and related conditions to address gaps in knowledge around drug use and build capacity around harm reduction and delivery of relevant evidence-based clinical interventions. The guidelines include an emphasis on people who use drugs who are at risk of experiencing harms relative to HIV, viral hepatitis and other related conditions.

As with critical areas within HIV, the social context, including social support, stigma and structural drivers such as employment, is important for health workers to understand. Harm reduction requires clinicians to understand the broader context in which drugs are used by their patients. The traditional ‘just say no’ approaches to drug use are as ineffective for drug use as they are for sex. Clinicians have an ethical obligation to their patients that extends to being advocates for evidence-based harm reduction.

Drug ‘harm reduction’ takes a pragmatic view that is humane, effective, holistic and fundamentally concerned with the rights of people who use drugs, their socio-economic context and the provision of services that are responsive, preventive and supportive. This approach also enhances the well-being of partners, family members and society at large.

Harm reduction approaches and related interventions are supported by a large body of evidence. Explicit support for needle-and-syringe services is included in the *South African National Strategic Plan for HIV, TB and STIs* (*2017–2022*), the *National Drug Master Plan* (*2019–2024*) and the *National Hepatitis Action Plan*. A National Department of Health policy around opioid substitution therapy and related clinical guideline is under development.

By integrating the guidelines in clinical practice, the quality of care provided by clinicians to people who use drugs will be enhanced – benefitting individuals and communities of people who use drugs and broader society.

## Scope and purpose of the guidelines

- Review evidence of the harm reduction approach
- Briefly review the epidemiology of drug use and its consequences
- Present clinical guidance for harm reduction interventions aligned with the framework developed by the World Health Organization
- Provide guidance around brief screening and interventions related to drug use
- For each harm reduction intervention, provide: a summary of evidence, main principles and links to related guidelines
- Highlight special considerations for young people who use drugs, women who use drugs, substance use and sexual encounters and drug use within prison settings
- Provide selected recommendations for stakeholders engaged in the delivery of harm reduction services in HIV, TB, viral hepatitis and related services.

## Audience

These guidelines are aimed primarily at clinicians (doctors, nurses and clinical associates). Other stakeholders who will benefit from this guideline include pharmacists, HIV and health programme officers and policymakers.

## Methods

A core writing team developed these guidelines. The process was informed by a review of evidence and guidance from the World Health Organization. A stakeholder consultation was held in August 2019, followed by international peer review. Inputs and recommendations were included.

## 1. Introduction

### 1.1 Harm reduction

People have always used drugs to alter health, perceptions, relationships and state of mind^1^ and this is not likely to change. Globally, in 2017, more than 271 million people had used unregulated drugs in the preceding year.^2^

The criminalisation of people who use certain drugs increases levels of stigma, encourages misinformation and contributes to harms, including high rates of preventable deaths.^3^ A purely biomedical approach – that presumes that all people who use drugs require treatment, and all drug use and dependence represents a disease requiring specialist medical intervention – carries the risk of stigma and often fails to pay due attention to the social and economic context in which dependent drug use occurs.^3^ Criminalisation and the pathologisation of drug use may intensify social disruption and hinder the provision of effective responses.^4^

Countries that have implemented comprehensive harm reduction programmes have managed to turn around epidemics of HIV and hepatitis C virus (HCV) infection. Overdose deaths are lower in contexts where harm reduction services are in place, compared with places where they are not. Harm reduction reduces the adverse health, social and economic consequences of drug use without necessarily reducing drug consumption.^5^ In 2018, 86 countries (11 in Africa) had at least one needle-and-syringe service and 86 (nine in Africa) had at least one opioid substitution therapy (OST) programme.

Harm reduction refers to policies, programmes and practices that aim to minimise negative health, social and legal impacts associated with drug use, drug policies and drug laws. Harm reduction is grounded in justice and human rights – it focuses on positive change and on working with people without judgement, coercion, discrimination, or requiring that they stop using drugs as a precondition of support. Harm reduction encompasses a range of health and social services and practices that apply to illicit and licit drugs. These include, but are not limited to, drug consumption rooms, needle and syringe programmes, non-abstinence-based housing and employment initiatives, drug checking, overdose prevention and reversal, psychosocial support and the provision of information on safer drug use. Approaches such as these are cost-effective, evidence-based and have a positive impact on individual and community health. (Harm Reduction International).^6^

#### Key points

- Harm reduction is an evidence, rights and public-health-based approach that reduces risks and improves the health and well-being of people who use drugs and the broader community.
- Long-term policies and interventions are needed to address structural factors that contribute to harms related to drug use.

BOX 1 South African policy.

South African health policy supports evidence-based interventions for people who use drugs. For example, the *South African National Drug Master Plan* (*2019–2024*) recommends access to the WHO-recommended package of comprehensive HIV prevention, treatment and care services for people who inject drugs. Similarly, the *South African National Strategic Plan on HIV, TB and STIs* (*2017–2022*) refers to the provision of harm reduction services, specifically OST and needle-and-syringe programmes. *The South African National Drug Master Plan* (*2019–2024*) includes these two interventions as part of the WHO-recommended package of services. The South African National Hepatitis Action Plan recommends access to viral hepatitis services that include access to harm reduction services for people who inject drugs.

TB, tuberculosis; STI, sexually transmitted infection; OST, opioid substitution therapy; WHO, World Health Organization.

Harm reduction principles for healthcare settings are listed.^7^

- **Humanism:** Care is given without moral judgement and with an understanding that choices are contextual.
- **Pragmatism:** The priority is the here and now, and the mitigation of immediate risk is what matters most.
- **Individualism:** People are different and have their own needs and strengths.
- **Autonomy:** People have a right to make informed choices, even against expert advice.
- **Incrementalism:** Any positive change is viewed as an improvement on current circumstances.
- **Accountability without termination:** People have the right to make choices, without their access to services being denied in relation to their decisions.

The application of these principles can improve patient–clinician relationships. The impact of harm reduction is increased through community engagement and peer-led services as well as removing barriers and increasing support.

#### Key points

- Harm reduction is an evidence, rights and public-health-based approach that reduces risks and improves the health and well-being of people who use drugs and the broader community.

### 1.2 Drugs and drug use

#### 1.2.1 Patterns of use

Depending on the drug, 8% – 15% of people who use drugs develop a problem with their use. Drug use occurs along a continuum and can shift according to various factors (see Table 1).

**TABLE 1:** Patterns of drug use.

Pattern of use	Description
Experimentation	Most young people will experiment with some activity that is outside of socially acceptable norms. Drugs are one of the ways people experiment. Most people will experiment for a limited period and then stop.
Non-dependent adult use	Many people consume alcohol in this way – it is used for social events, and largely remains non-problematic. Many people will use unregulated drugs in the same way.^8^
Conscious, regulated use	Many drugs are used only in certain ways and circumstances, according to a set of cultural or individual rules and accepted norms. Cannabis use by Rastafarians is an example, as is the planned use of hallucinogenic drugs to find answers to a specific problem. When someone makes a well-informed conscious choice to use a specific drug in a specific way in specific circumstances, it will seldom become problematic.
Dependence	The International Classification of Diseases and Related Health Problems (ICD) version 11 (ICD-11) defines this as ‘a disorder of regulation of [specific drug] use arising from repeated or continuous use of [specific drug]. The characteristic feature is a strong internal drive to use [specific drug], which is manifested by impaired ability to control use, increasing priority given to use over other activities and persistence of use despite harm or negative consequences. These experiences are often accompanied by a subjective sensation of urge or craving to use [specific drug]. Physiological features of dependence may also be present, including tolerance to the effects of [specific drug], withdrawal symptoms following cessation or reduction in use of [specific drug, notably with opioids], or repeated use of [specific drug] or pharmacologically similar substances to prevent or alleviate withdrawal symptoms. The features of dependence are usually evident over a period of at least 12 months but the diagnosis may be made if [specific drug] use is continuous (daily or almost daily) for at least 1 month.’^9^
Habituated use, commonly called addiction	When someone appears to have little control over their drug use and they have learned to use drugs as the automatic response to problems they face or this use is how they can feel alive and engaged, such use can become problematic to the individual and those around them. Habituated drug use is also often dependent. This form of use is commonly called ‘addiction’. Addiction applies to an all-consuming relationship with a drug, person or activity to the detriment of the individual.

#### 1.2.2 Methods of drug use

Drugs can be taken by different administration modes, which can lead to different effects and varying degrees of harm. For instance, intravenous (IV) administration is associated with rapid onset and peak of action, with elevated risks: for opioids, this includes overdose and for stimulants, such as cocaine, this includes arrhythmia. Common methods of drug use are summarised here.

- **Smoking** is the most common form of use for cannabis, methaqualone (*mandrax*), heroin (*whoonga, nyaope, sugars*) and methamphetamine (*tik, crystal meth*) in southern Africa. Onset of action is faster than other forms of use. Risks are related to airways and pulmonary disease.
- **Nasal inhalation** (*snort, schnarf, toot, sniff*) is often used for cocaine, but also heroin. Onset of action is quick and is associated with risk of damage to the nasal mucosa.
- **Injecting** (*slam, spike, smoke*) can be done through several routes, most commonly IV. Heroin is the most injected drug, followed by methamphetamine and cocaine. Onset of action is very rapid. Risks are largely related to the use of contaminated injecting equipment and poor hygiene practices, including local and blood-borne infections (notably HIV, hepatitis B virus [HBV] and HCV). The risk of overdose is higher if drugs are injected. A proportion of people who use opioids and/or stimulants for a long period of time will transition to injecting.
- **Oral ingesting** (*pop*) is the most common route for gamma-hydroxybutyric acid (GHB), alcohol, methadone and ecstasy, amongst others. Onset is slower and risks may vary depending on the food or liquids consumed.
- **Rectal suppository** or **vaginal** (*booty bumping*) administration are less frequent methods of substance intake.

#### 1.2.3 Drug classifications and common drugs

Drugs can be categorised into five broad classes according to their primary effects: stimulants, depressants, hallucinogens, cannabinoids and antipsychotics. An overview of common drugs is given in Table 2.

**TABLE 2:** Overview of common drug types.

Category	Description	Common examples
Stimulants	Also known as ‘uppers’, these drugs increase energy.	Caffeine, cocaine, methamphetamine (*tik*), methcathinone, ecstasy, methylphenidate†
Depressants	Also known as ‘downers’, these drugs decrease brain activity and tend to have a calmative effect, making people feel relaxed and drowsy and sometimes leading to a state of lucid dreaming	Sedative hypnotics (e.g. alcohol, barbiturates and methaqualone [mandrax ]), narcotic analgesics (morphine), heroin (*nyaope*/*whoonga*/*sugars*), benzodiazepines and GHB
Hallucinogens	A diverse group of natural and synthetic drugs that alter consciousness, perception, thinking and can cause auditory and visual hallucinations.	Lysergic acid diethylamide (LSD), psilocybin (magic mushrooms), 3,4-methylenedioxymethamphetamine (MDMA, *ecstasy* or *XTC*) and ketamine
Cannabinoids	The flowering head of the cannabis plant. The flowers can be processed into resins (e.g. hashish) and oils or dried and smoked or turned into edibles. It is used to treat nausea, pain, loss of appetite and spasticity. Cannabidiol (CBD) can also act as an antipsychotic,^10^ whilst tetrahydrocannabinol (THC) can increase the move towards psychosis for people with a vulnerability to psychosis (the relationship has not been proven to be causative).	CBD and THC
Antipsychotics	Used mainly for the treatment of psychotic disorders. They are seldom used outside a medical setting as there are unpleasant side effects that outweigh the benefits for people without a diagnosis of a psychotic disorder.	Quetiapine, olanzapine and risperidone

CBD, cannabidiol; GHB, gamma-hydroxybutyric acid; LSD, lysergic acid diethylamide; MDMA, 3,4-methylenedioxymethamphetamine; THC, tetrahydrocannabinol.

† , Methamphetamine and methcathinone are part of the large group of amphetamine-type stimulants.

#### 1.2.4 Epidemiology of drug use in southern Africa

As a result of the illegal nature of drug use and associated stigma, obtaining robust data on drug use is difficult and data are limited (see Table 3). It is therefore likely that estimates of reported use and risks are under-reported. Research reflects increased trafficking of heroin in the region and a review of substance use treatment data in South Africa points to a six-fold increase in heroin-related admissions over the last decade, with marked increases seen in methamphetamines and other stimulant-related admissions during the same period.^13^

BOX 2 Myths about drug use.

An excellent resource on myths about drugs and drug use is available at: https://www.changingthenarrative.news/. Common myths are described in detail in Appendix 1.

BOX 3 Heroin has many names.

*Whoonga, nyaope, sugars* and *unga* are all heroin-based drugs.^11^ They may have ‘cutting’ agents (e.g. pharmaceutical opioids, caffeine and inactive powders), which are used to decrease the strength of the drug and bulk up the volume to increase profit. Contrary to popular belief, few tested samples of *nyaope, whoonga* or *unga* have contained any traces of antiretroviral medication.^12^

BOX 4 Image and performance-enhancing drugs.

Anabolic steroids, peptides and hormones are examples of drugs that may be linked to appearance, the pursuit of health and youth, or a body image disturbance. Patterns of use may vary, with potential health risks related to the substances used, as well as the method of use (e.g. safe injecting or not).^11^

BOX 5 Drug, (mind-) set and setting.^8^

The effect of a drug is not a predictable chemical cascade. Even when the same person takes the same drug, in the same dose, the results may differ depending on a number of variables.^8^ Drug effect is mediated by the drug itself, the biology and mindset of the person taking the drug and the setting and context in which the drug is taken.^8^ For example, heroin bought from a street dealer usually has a different effect to diamorphine given by doctor in a medical setting, even though they are the same drug.

**TABLE 3:** Overview of substance use epidemiology (latest data).^14,15,16,17^

Drug	Prevalence of use (%) (15–64 years)	Treatment† (%) (July–December 2018)	Comments
**South Africa**			
Heroin	0.3 – 0.5^14,17^	19 (2 – 34)^15^	Prevalence of use reflects use for different recall periods. The types of substance used vary by region.
Cocaine	1^14^	3 (2 – 8)^15^	
Methamphetamine	1^14^	10 (1 – 28)^15^	
Cannabis	4^14^	30 (22 – 38)^15^	
Ecstasy	ND	< 0.5^15^	
**Botswana**			
Opioids	< 0.05^16^	ND	Cross-sectional survey amongst first-year university students, reflecting use in previous year.
Cocaine	< 1^16^	ND	
Amphetamine-type stimulants	8^16^	ND	
Cannabis	9^16^	ND	
Ecstasy	< 1^16^	ND	

ND, no data.

† , Reflects proportion of admissions for primary substance at treatment centres registered with the South African Community Epidemiology Network on Drug Use. Data are presented as median and range across regions of the country.

#### 1.2.5 Drug-related harms

The harms related to drugs are embedded in social and structural circumstances, including stigma, which is often driven by the illicit nature of drug use. For a variety of reasons, the production, sale and use of certain drugs are restricted or highly regulated through international agreements.^18^ People who use drugs, especially those who inject drugs, are vulnerable to several health issues including HIV, viral hepatitis, cellulitis and infective endocarditis.^19^ People who use drugs are also at increased risk of developing tuberculosis (TB).^20^ Long-term smoking of drugs (including cannabis, methaqualone or heroin), particularly amongst people who also smoke tobacco products, can increase risks for the development of chronic obstructive airways disease and emphysema.^21,22^ Globally, the incidence of HIV infection has declined, yet infections amongst people who use drugs continue to increase.^23^

The quantification of drug-related harms in South Africa, including HIV and viral hepatitis infections and overdose, is inadequate. Table 4 provides a snapshot of available data.

BOX 6 Drug scheduling.

The schedule of a drug determines the level of regulation and access. The WHO Expert Committee on Drug Dependence provides recommendations based on rigorous scientific review to the CND, which then decides on drug scheduling.^29^ Scientific approaches to assess the relative risks of drugs do not inform the scheduling decided by the CND. The scheduling of drugs by the CND has historically been based on political agendas. For example, cannabis, LSD and MDMA are in the ‘most dangerous’ schedule, but are linked to low levels of harm.^30^

CND, Commission on Narcotic Drug; LSD, lysergic acid diethylamide; MDMA, 3,4-methylenedioxymethamphetamine; WHO, World Health Organization.

BOX 7 Restricting access can render drugs more dangerous.

Drugs are influenced by market forces, with supply developed to meet a demand. Restricting access to potentially harmful drugs can make them more dangerous. Quality control is often absent in the illicit drug trade. For example, although diamorphine and heroin are the same drug, street heroin is twice as dangerous as morphine.^30^ That risk increases significantly if the heroin is contaminated with a stronger opioid like fentanyl. Furthermore, bulking agents are used to increase the volume of drugs to increase profit, some of which can cause harm.

**TABLE 4:** Overview of infectious disease prevalence and morbidity amongst people who inject drugs (latest data).

Health issue	Mozambique (%)	South Africa (%)	Comments
HIV	46^24^	21 (8 – 56)^25,26^	HIV prevalence estimates amongst people who inject drugs in South Africa from major metropolitan areas
TB	ND	ND	
STIs	8 – 29^24^	ND	Self- reported genital ulcer
HCV	67^24^	55^26^	
HBV	32 – 36^24^	5^26^	
HIV–HCV coinfection	12 – 45^24^	9 – 57^25^	Includes HBV or HCV co-infection
Number of drug-related deaths	ND	10^17^	Ten drug-related deaths included in the formal surveillance system, reported in 2012
Mental health	ND	ND	Between a quarter and half of patients with an opioid use disorder in public in-patient substance use treatment centres have been found to have co-occurring mental illness^27,28^

HBV, hepatitis B virus; HCV, hepatitis C virus; STIs, sexually transmitted infections; TB, tuberculosis; ND, no data.

#### 1.2.6 Reasons for drug use

The reasons why people are using drugs outside of supervised medical care are poorly understood. The dominant discourses to explain this phenomenon are often based on moral or political foundations rather than science. It is beyond the scope of these guidelines to provide a comprehensive analysis of the use of drugs. People use drugs for a range of reasons (Table 5),^31^ and many myths exist in the context of clinical care around people who use drugs (see Appendix 1).^32^

**TABLE 5:** Insights into reasons for drug use amongst people with unstable housing, South Africa (2015).^31^

Reasons	Comments
Trauma and loss	‘I grew up in an abusive family, being sexually abused by my grandfather and having alcoholic parents.’ ‘I lost my husband and the will to live. Instead of killing myself outright I was slowly killing myself with the taking of drugs and once again to numb the pain and to forget what I had lost in my life.’
Exclusion from society and connection with other people who use drugs	‘When my family rejected me because of my gender of being a transwoman, I started hanging out with people who used.’ ‘The community calls us names and we are [*a*] disgrace to the society, people are very judgemental of us.’
Self-preservation and self-medication	‘I have got to the point where I no longer consider what I am using to be drugs – it is now a medicine.’ ‘I do not feel well or function properly unless I have had heroin. I need it to fuel my creativity.’ ‘It keeps me warm on the street. It keeps me awake for my service that I deliver as a transgender sex worker.’

Chronic problematic drug use is largely caused by personal, social, cultural and political pain and suffering (and at times may also include psychological, physiological and legal issues).^33^

BOX 8 Overlapping vulnerabilities and intersectionality.

Many people engage in multiple activities that may increase their risk of being exposed to HIV, STIs and viral hepatitis, as well as onward transmission of these infections (e.g. someone who injects drugs and also sells sex, or a man who has sex with other men and uses drugs in the context of sexual encounters). Furthermore, some of the vulnerabilities that are related to social constructs may interact and compound risk. Clinicians should avoid stereotyping people and openly enquire about gender identity, as well as sexual and drug-using practices.

STIs, sexually transmitted infections.

BOX 9 Harm reduction case studies.

**Mauritius:** In the 2000s, Mauritius had one of the worlds’ highest levels of opiate use, and the HIV epidemic is highly concentrated amongst people who inject drugs. In 2006, it was the first African State to launch a needle-and-syringe service and provide OST. Services have expanded and include OST provision within prison. In 2020 there are 47 needle-and-syringe services sites (36 operated by the government and 11 by civil society) with around 3000 clients. There are 44 OST sites (12 in Area Health Centres, 4 in prisons and 28 outside of police stations), servicing 5300 people.^34^ HIV incidence amongst people who inject drugs decreased from 92% in 2005 to 31% in 2014.^35^ Opioid substitution therapy has enhanced the quality of life, family environment and self-esteem of OST patients. Moreover, during the same period, a decrease in criminality rates was observed, from 2650 cases in 2007 to 1085 in 2012.^36^

**Kenya:** Kenya is recognised as a regional leader in the implementation of harm reduction. Needle-and-syringe services were provided in five pilot sites between 2012 and 2015 and saw a four-fold reduction in needle-sharing practices (from 48% to 12%), drastically reducing HIV incidence amongst people who inject drugs.^34^ Methadone has been available since 2014, and by 2018, over 2000 people across seven treatment sites were receiving OST.^5^ Programmes provide holistic HIV prevention and treatment, SRH and mental health services for people who use drugs.

*Source:* Mauritian case study developed by Kunal Naik; Kenyan case study developed by Bernice Apondi

OST, opioid substitution therapy; SRH, sexual and reproductive health.

#### 1.2.7 The relevance of harm reduction

It will take a long time to affect a significant reduction in the number of drugs used, and the harms caused because this requires structural reform, which is explored later. The historical focus on abstinence and law enforcement has been ineffective and resulted in significant harm. Harm reduction is an effective public health intervention. It keeps people alive and reduces drug-related morbidity. For individuals, harm reduction aligns with the tenets of medical ethics in that it is beneficent and patient centred. It takes a longer-term view and helps people meet their goals in a stepwise manner. Specific harm reduction interventions relevant to people who use drugs are covered in the section ‘Evidence-based interventions’.

#### Key points

- People use drugs for many reasons, in an array of circumstances and contexts and this cannot be modified rapidly.
- Not all drug use is harmful.
- Addressing drug use in isolation will seldom result in a sustained resolution, unless the underlying motivators are addressed.
- People use drugs in different ways; drugs are mostly smoked in South Africa, but injecting is becoming more prevalent.
- Drug-related risks and effects depend on the drug, (mind-)set and setting.
- The use of opioids and amphetamine-type stimulants in the region is increasing.
- In the context of criminalisation, many people who use drugs enter and exit the criminal justice system, placing them at risk for HIV and other infectious diseases.
- The burden of HIV, viral hepatitis and TB amongst people who use drugs in the region is high.

#### 1.3 Stigma, discrimination and human rights

Stigma is a process of exclusion; it occurs when a person – or group of people – are tainted or disgraced. When people perceive themselves as being stigmatized, they may also come to hold the same negative perceptions about themselves, leading to an internalization of stigma and acceptance of a ‘spoiled identity’.^37^

Stigma, misinformation and the lack of evidence-based harm reduction approaches are major contributing factors to the vulnerabilities people who use drugs face. Stigma is often not prioritised by healthcare professionals, yet has a profound effect on the relationships between clinicians and their patients.^38^

The use of non-stigmatising language can enhance relationships with patients and clinical outcomes. Table 6 outlines alternative supportive language to use.

**TABLE 6:** The use of non-stigmatising language to enhance patient outcomes.^40^†

Principles	Recommended wording to use	Wording to avoid
Separate the person from the behaviour Do not use pejorative terms as a noun Avoid words with a moral or negative connotation Do not use language that vilifies or separates people	*People who use drugs* *A person who uses* [alcohol] *People who are dependent on drugs* *People who inject drugs*	*A user* *Addict* *Alcoholic* *Junky* *Injector*
	*They no longer use …* *They have chosen to abstain from [heroin]* *They stopped using cocaine* *They resolved their dependent heroin use*	*Clean* *Dirty*
	*Substance/drug use*	*Substance abuse*

† , Additional information and responses around drug use and substance use disorders and treatment can be found at: https://www.changingthenarrative.news/.

‘Stigma in health facilities undermines diagnosis, treatment, and successful health outcomes. Addressing stigma is fundamental to delivering quality healthcare and achieving optimal health.’^39^

The International Network of People Who Use Drugs (INPUD) recognises that language cannot be regulated, and that context can transform a term that is used to oppress into one through which emancipation is pursued … Ordinarily, however, language that may denigrate, is best avoided.^40^

Discrimination, which is the enactment of stigma, also needs to be addressed and the rights of all people secured. Many governments in the region have signed the *International Covenant on Economic, Social and Cultural Rights,*^40^ which outlines the range of rights that are relevant to people who use drugs in health settings. Some of the relevant rights are: the right to self-determination (Article 1); the right to non-discrimination based on race, colour, sex, language, religion, political or other opinion, national or social origin, property, birth or other status (Article 2); and the right to enjoy the highest attainable standard of physical and mental health (Article 12). People who use drugs experience frequent violation of these rights, which increases the harms of drug use – including confiscation of sterile injecting equipment and medication that forms part of substance use disorder treatment or other health conditions.^41^

#### Key points

- People who use drugs frequently experience stigma, discrimination and human rights violations, which negatively affect their health and well-being.
- The use of appropriate language is an important component of providing support services.

### 2. Evidence-based interventions

#### 2.1 A guiding framework

These guidelines are built upon the framework set out in the WHO *Consolidated Guidelines on HIV Prevention, Diagnosis, Treatment and Care for Key Populations*,^19^ including health sector interventions (Table 7) and critical enablers (Table 8).

**TABLE 7:** Health sector interventions.^19^

Variable	Description
HIV prevention	The correct and consistent use of condoms with condom-compatible lubricants. PrEP should be offered as an additional prevention choice for key populations at substantial risk of HIV infection. PEP should be available to all eligible people from key populations on a voluntary basis after possible exposure to HIV. VMMC should be promoted as an additional efficacious HIV prevention option within combination HIV prevention packages for adolescents aged ≥ 15 years and adult men in settings with generalised epidemics, to reduce the risk of heterosexually acquired HIV infection.
Harm reduction for people who use drugs	All people who inject drugs should have access to sterile injecting equipment through needle-and-syringe services. People who are dependent on opioids should be offered and have access to OST. People with harmful alcohol or other substance use should have access to evidence-based interventions, including brief psychosocial interventions involving assessment, specific feedback and advice. People likely to witness an opioid overdose should have access to naloxone and be instructed in its use for emergency management of suspected opioid overdose. Provide interventions that support harm reduction for people who use stimulants, provide safe spaces for drug consumption and means for drug checking.†
HIV testing and counselling	Voluntary HIV testing and counselling should be routinely offered to all key populations, both in the community and in clinical settings. Community-based HIV testing and counselling for key populations, linked to prevention, care and treatment services, is recommended, in addition to PITC.
Treatment and care	All people living with HIV should have the same access to ART and ART management. All pregnant women should have the same access to services for PMTCT and follow the same recommendations.
Prevention and management of coinfections and comorbidities	People who use drugs should have the same access to TB prevention, screening and treatment services as other populations at risk of or living with HIV. People who use drugs should have the same access to HBV and HCV prevention, screening and treatment services as other populations at risk of or living with HIV. Routine screening and management of mental health disorders (especially depression and psychosocial stress) should be provided for people from key populations living with HIV to optimise health outcomes and improve their adherence to ART.
Sexual and reproductive health	Screening, diagnosis and treatment of STIs should be offered routinely as part of comprehensive HIV prevention and care for key populations. People from key populations, including those living with HIV, should be able to experience full, pleasurable sex lives and have access to a range of reproductive options. Abortion laws and services should protect the health and human rights of all women. It is important to offer cervical cancer screening to all women who use drugs. It is important that all women who use drugs have the same support and access to services related to conception and pregnancy care, as women from other groups.

ART, antiretroviral therapy; PMTCT, prevention of mother-to-child transmission of HIV; PEP, post-exposure prophylaxis; PrEP, pre-exposure prophylaxis; STIs, sexually transmitted infections; TB, tuberculosis; VMMC, voluntary medical male circumcision; OST, opioid substitution therapy; PITC, provider-initiated testing and counselling; HBV, hepatitis B virus; HCV, hepatitis C virus.

† , These interventions are not part of the WHO Framework, but evidence in support of these harm reduction interventions is increasing. Drug checking is most useful to detect contamination with/presence of potent synthetic opioids.

**TABLE 8:** Critical enablers.^19^

Number	Description
1.	Laws, policies and practices should be reviewed and, where necessary, revised by policymakers and government leaders, with meaningful engagement of stakeholders from key population groups, to allow and support the implementation and scale-up of healthcare services for key populations.
2.	Countries should work towards implementing and enforcing antidiscrimination and protective laws, derived from human rights standards, to eliminate stigma, discrimination and violence against people from key populations.
3.	Health services should be made available, accessible and acceptable to key populations, based on the principles of medical ethics, avoidance of stigma, non-discrimination and the right to health.
4.	Programmes should work towards implementing a package of interventions to enhance community empowerment amongst key populations.
5.	Violence against people from key populations should be prevented and addressed in partnership with key population-led organisations. All violence against people from key populations should be monitored and reported and redress mechanisms should be established to provide justice.
6.	Appropriate funding should be made available to support harm reduction services.†

† , This enabler is not part of the WHO Framework, but critical for sustainability and impact.

#### 2.2 Assessing a person’s needs

The screening for substance use and offer of assistance for potentially harmful substance use can take place in a range of clinical scenarios (see Appendix 2). The integration of screening for substance use and mental health conditions, linked to brief interventions and referral for further treatment, is often the first step in supporting people within a harm reduction approach and is outlined here.

#### 2.2.1 Screening and brief intervention for common mental disorders and harmful substance use

Substance use disorders fall into the category of common mental disorders. Harmful alcohol and other drug use and other mental health disturbance may result in an increased risk of contracting HIV and in substantial health problems amongst people living with HIV. However, it is important to note that most people who use drugs do so on an occasional basis and will not develop a substance use disorder (dependence). For this group, there may be little need for high-intensity interventions. Screening for other common mental disorders including depressive disorders and anxiety disorders should also be performed. These may arise because of psychosocial distress that may be related to the HIV diagnosis or as a consequence of infective processes, which may be primary or secondary in a patient living with HIV. Identification of these is essential as they may impact on clinical outcomes and capacity to adhere to ART. Appropriate treatment is likewise freely available, so there is no need for patients to suffer. Suicidal screening should form part of this assessment because of its association with common mental disorders and the particularly high risk within this population.^42^

**FIGURE 1:**
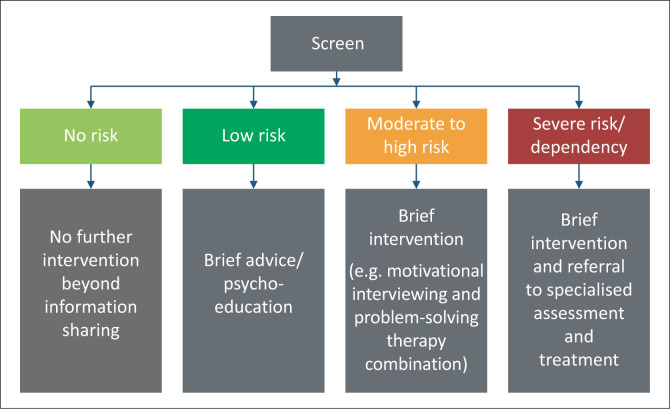
Pathways following screening for harmful substance use.^50^

**Screening, brief intervention and referral to treatment (SBIRT)** for harmful substance use is an evidence-based approach to improve the detection and early intervention of harmful substance use to prevent or address dependence.^43^ The three core components of SBIRT are (1) universal screening, followed by (2) risk triaging, to determine (3) the appropriate level of intervention and/or referral to specialty assessment and care (Figure 1).

**2.2.1.1 Screening:** Screening people at risk for, or living with, HIV for harmful alcohol and/or drug use is crucial and can be performed in a myriad of settings including consulting rooms, emergency units, hospital wards and community settings (see Appendix 2). Screening tools such as the Alcohol, Smoking and Substance Involvement Screening Test (ASSIST)^44^ and Alcohol Use Disorders Identification Test (AUDIT) make use of risk categories determined by screening scores to help determine the ideal intervention strategy (Table 9). Additional alternative validated tools are listed in Appendix 2.

**TABLE 9:** Alcohol, Smoking and Substance Involvement Screening Test risk score and associated risk level and intervention.^45^

Alcohol	All other substances†	Risk level	Intervention
0–10	0–3	Lower risk	• General health advice
11–26	4–26	Moderate risk	Brief intervention Take-home booklet and information
≥ 27	≥ 27	High risk	Brief intervention Take-home booklet and information Referral to specialist assessment and treatment
Injected drugs in last 3 months‡		Moderate and high risk	Information card on risks of injecting Brief intervention Take-home booklet and information Referral to testing for BBVs§ Referral to specialist assessment and treatment

BBVs, blood-borne viruses; HBV, hepatitis B virus; HCV, hepatitis C virus.

† , Tobacco products, cannabis, cocaine, amphetamine-type stimulants, sedatives, hallucinogens, inhalants, opioids and other drugs.

‡ , Need to determine pattern of injecting – injecting more than four times per month (average) over the last 3 months is an indicator of dependence requiring further assessment and treatment.

§ , Blood-borne viruses including HIV, HBV and HCV.

**2.2.1.2 Brief interventions:** A brief intervention is a short (time-limited), often opportunistic, patient-centred strategy, where a healthcare provider provides targeted information and/or advice to individuals during the course of other health activities such as routine outpatient review or HIV testing.^45^ The aim of the interaction is to increase insight and awareness of harmful substance use to facilitate a patient’s motivation to modify risky behaviour. Brief interventions thus seek to reduce drug use and associated behaviours, which increase the risk of contracting or transmitting HIV, for example, risky sexual behaviour and unsafe drug injecting practices. There is little difference in the outcomes between longer, more intensive interventions and brief interventions; and brief interventions are practical, cost-effective and have a growing evidence base.^46,47^

Behavioural interventions, self-regulation coaching and psychosocial counselling can support HIV harm reduction and other HIV prevention objectives for people who use substances, whilst also contributing to longer-term and broader health and wellness goals.^48,49^ Brief interventions should be provided to people with moderate-to-high risk and above substance use. Clinical guides or steps for the use of common interventions follow, with details in Appendix 2.

BOX 10 Key components of brief interventions.^50^

1. Providing information and feedback about screening results
2. Understanding the patients’ views of their use and then coaching the patients to change their perceptions about their use
3. Encouraging the patients to discuss their views on how their use led to their injury, their likes and dislikes about use and how they may consider changing
4. Advising patients in clear but respectful terms to decrease or abstain from substances
5. Teaching behaviour change skills that will reduce substance use as well as the chances of negative consequences
6. Establishing a method for follow-up with the patient; follow-up can be done in another visit or telephonically

BOX 11 Motivational interviewing and adherence.^51^

Brief interventions based on the principles of motivational interviewing (MI), with the possible addition of other complementary approaches such as contingency management (CM), can reduce drug-related high-risk sexual behaviours, increase adherence to ART and maximise PrEP amongst patients who are dependent on stimulant drugs.^51^

ART, antiretroviral therapy; CM, contingency management; MI, motivational interviewing; PrEP, pre-exposure prophylaxis.

Elements of brief interventions may be aligned to the stages as outlined in Table 10. It is important to remain mindful of patient’s social and economic context, an element some reviewers have flagged as not necessarily accounted for by strictly following these stages.

**TABLE 10:** Stages of change and recommended brief intervention elements.

Stage	Definition	Brief intervention elements to be emphasised
Pre--contemplation	The hazardous or harmful alcohol and/or drug user is not considering change soon, and may not be aware of the actual or potential health consequences of continued risky alcohol and/or drug use	Feedback about the results of the screening and information about the hazards of continued risky alcohol and/or drug use
Contemplation	The alcohol and/or drug user may be aware of alcohol and/or drug-related consequences but is ambivalent about making a change	Emphasise the benefits of making a change, give information about problems related to risky alcohol and/or drug use, including the risks of delaying change and discuss how to choose a goal
Preparation	The alcohol and/or drug user has already decided to make a change and plans to act	Discuss how to choose a goal, and give advice and encouragement
Action	The alcohol and/or drug user has begun to cut down or reduce risky alcohol and/or drug use, but change has not become a permanent feature	Review advice, give encouragement
Maintenance	The alcohol and/or drug user has achieved moderate drinking/drug use or abstinence on a relatively permanent basis	Give encouragement and support as required or requested

**2.2.1.3 Referral for treatment:** People with severe risk/dependency, as identified by a screening tool, require additional and more intensive support. If these are not provided by the person conducting the screening, then the patient should be referred for further assessment and management by a substance use disorder specialist at an appropriate facility.

**TABLE 11:** Who should screen, provide brief interventions for substance use and refer for care.

Healthcare worker	Patient encounters and potential scope of work
Doctors, nurses, CAs, CHWs, pharmacists, psychologists, counsellors, social workers	Consultations, harm reduction counselling
Nurses, CAs	Case management of patients on OST, management of HIV and TB treatment, managing overdose, case management of HBV and HCV
Nurses, CAs, CHWs	Case management of patients on HIV, TB, viral hepatitis treatment, managing overdose
Doctors, nurses, CAs	Integrated service delivery, management of HIV and TB, managing overdose
Doctors	Prescribing and managing patients on OST, withdrawal management, management of viral hepatitis
Pharmacists (community- and facility-based) psychologists, counsellors, social workers, CHWs	Supporting patients on OST, HIV, TB, viral hepatitis treatment; needle-and-syringe service
Paramedics	Managing emergencies
All (including allied health professionals and traditional health practitioners)	Screening, brief interventions and referral for medical and psychosocial services

CAs, clinical associates; CHWs, community health workers; HBV, hepatitis B virus; HCV, hepatitis C virus; OST, opioid substitution therapy; TB, tuberculosis.

### 2.3 HIV prevention

#### 2.3.1 Condoms and lubricant

**TABLE 12:** HIV prevention – condoms and lubricant.^19^

Variable	Description
Evidence and implementation experience	The correct and consistent use of male condoms reduces the risk of HIV transmission through anal and vaginal sex by 94% and prevents unintended pregnancy and common STIs. Condom-compatible lubricant (i.e. silicone- or water-based) reduces breakage and slippage. Condoms and lubricant are widely included in harm reduction commodity distribution services – for people who inject drugs, people who use stimulants and for women who use drugs.
Main principles	A sufficient quantity of condoms and lubricants (one-to-one ratio) along with counselling around safer sexual practices must form part of the comprehensive harm reduction package for people who use drugs and their sexual partners.^19^
Guidelines	Comprehensive condom programming – a guide for resource mobilisation and country programming: https://www.unfpa.org/sites/default/files/pub-pdf/CCP.pdf

STIs, sexually transmitted infections.

#### 2.3.2 Pre- and post-exposure prophylaxis

**TABLE 13:** HIV prevention – pre- and post-exposure prophylaxis.^19^

Variable	Description
Evidence and implementation experience	There is strong evidence showing that, amongst those who are adherent, PrEP is highly effective in reducing HIV transmission through sexual exposure.^19^ Evidence supporting a reduction in parenteral transmission is not as strong, nor is it supported by real-world implementation. In order for PrEP to be effective, it must be taken for a minimum of 7 days before potential exposure to HIV. In addition, it is recommended that PrEP be taken for 28 days after the last potential exposure.
Main principles	The priority HIV prevention interventions for people who inject drugs remain access to sterile injecting equipment and OST for those who have an opioid dependency. PrEP and PEP are integral parts of packages of care for people who use drugs. PrEP should be offered to people who inject drugs who are interested in PrEP, do not have any contraindications to use, and are at substantial risk for HIV, defined as: - HIV-negative people who inject drugs with HIV-positive/unknown status injecting and/or sexual partner(s) - Sharing injecting needles and/or drug preparation equipment - People who use/inject drugs and also have risk because of sexual transmission - Sexual partners of people who use/inject drugs PEP is the only way to reduce the risk of HIV infection after exposure. PEP should be administered ≤ 72 h after exposure in order for it to be effective, and a full 28 days is required after exposure for full protection. PEP should preferably consist of a combination of tenofovir disoproxil fumarate (TDF) + lamivudine (3TC) or emtricitabine (FTC) and an integrase strand transfer inhibitor (InSTI). Women should be provided with contraception (with due consideration of relevant drug–drug interactions). The regimen for PrEP is most commonly a combination of TDF and FTC. New PrEP options, including injectables, are under development. Requests for PEP should immediately trigger a conversation as to whether the individual should transition to PrEP after completing the 28 days of PEP. Discussions should explore sexual- and drug-use-related risks and use of sterile injecting equipment if appropriate. Regular HIV testing, assessment for HBV, HCV and renal function, and adherence support should all be routine components of PEP and PrEP service provision. Neither PrEP nor PEP is contraindicated for persons with HBV. However, before deciding to stop PrEP or PEP, they should discuss with their provider to avoid experiencing a hepatitis flare. Requests for PrEP and/or PEP should be addressed urgently and diligently, and should be seen as an opportunity to provide an expanded package of care for people who use drugs and allow for access to other important services. These include access to sterile injecting equipment, HIV testing services and other risk reduction interventions. PrEP and PEP only provide protection against HIV. They do not protect against other STIs or other associated health risks that can be addressed by expanded access to care.
Guidelines	Southern African guidelines on the safe use of PrEP in persons at risk of acquiring HIV-1 infection: https://sahivsoc.org/Files/Guidelines on the safe use of PrEP (March 2016).pdf Southern African guidelines on the management of occupational and non-occupational exposure: https://sahivsoc.org/Files/Guideline on the management of occupational and non-occupational exposure (PEP) (Mar 2016).pdf

3TC, lamivudine; FTC, emtricitabine; InSTI, integrase strand transfer inhibitor; PEP, post-exposure prophylaxis; PrEP, pre-exposure prophylaxis; STIs, sexually transmitted infections; TDF, tenofovir disoproxil fumarate; HBV, hepatitis B virus; HCV, hepatitis C virus; OST, opioid substitution therapy.

#### 2.3.3 Voluntary medical male circumcision

**TABLE 14:** HIV prevention – voluntary medical male circumcision.^19^

Variable	Description
Evidence and implementation experience	VMMC reduces the risk of female-to-male HIV transmission by 60% and is recommended as an additional prevention intervention for heterosexually acquired HIV infection in men.^19^
Main principles	VMMC should continue to be promoted as an additional efficacious HIV prevention option within combination HIV prevention packages for adolescents aged ≥ 15 years and for adult men in settings with generalised epidemics to reduce the risk of heterosexually acquired HIV infection.^19^
Guidelines	South Africa national guidelines for medical male circumcision: https://www.usaidassist.org/sites/default/files/sa_mmc_guidelines.pdf

VMMC, voluntary medical male circumcision.

### 2.4 Harm reduction interventions

#### 2.4.1 Needle-and-syringe services

**TABLE 15:** Harm reduction interventions – needle-and-syringe services.

Variable	Description
Evidence and implementation experience	Needle-and-syringe services, also known as needle-and-syringe programmes, are structured services that allow people who inject drugs to obtain new, sterile needles and syringes and other injecting equipment (including sterile water, alcohol swabs, tourniquets, cookers, etc.) at little or no cost, in order to reduce the risk of HIV and HCV infection.^52^ They also include mechanisms for the safe return and destruction of used needles and syringes and other injecting equipment. Needle-and-syringe services are the cornerstone of the HIV and viral hepatitis response for people who inject drugs.^23^ These services: - reduce sharing of needles and unsafe injecting practices by up to 60% - decrease HIV transmission by up to 33% – 42%^53,54^ - increase contact of healthcare workers with people who inject drugs who would not otherwise access health services^5^ - in the context of high coverage, and in combination with OST, reduce HCV transmission - are some of the most cost-effective public health interventions to date^55,56^ - do not increase rates of people starting to inject, nor do they increase frequency of injecting or increase drug use^19^ - do not decrease motivation to reduce or stop drug use.^54,57,58,59^ In 2018, needle-and-syringe services were available in 86 countries.^5^ For viral hepatitis elimination, the WHO’s target is 300 needle sets to be distributed per people who inject drugs per year by 2030.^60^ However, the increased circulation of needles and syringes can raise concerns. Occupational needle-stick injuries amongst police during searches and operations are of major concern.^61,62,63^ Injecting equipment discarded in public spaces may spark public alarm, with complaints often directed to the police. Needle-and-syringe services address these concerns by providing people who inject drugs with the tools and skills required to prevent blood-borne infections^64^ and increasing access to mechanisms for the safe collection and disposal of used injecting equipment, thereby protecting the public and emergency/other workers from needle-stick injuries.^52,65,66,67,68^ Needle-and-syringe services are a crucial gateway to HIV and other services, such as OST, HIV testing and counselling and treatment for HIV, TB and viral hepatitis.^5^
Main principles	Denying access to harm reduction services and confiscating injecting equipment have negative health and safety outcomes. These actions increase the likelihood of needle-and-syringe concealment, reuse, sharing and unsafe disposal, increasing the risk of HIV and blood-borne infection transmission to people who inject drugs, the police and general public.^69,70,71,72^ People who inject drugs should have access to enough injecting equipment to allow for a new needle/syringe to be used with each injection, along with sterile water and alcohol swabs. Additional materials that reduce risk include cookers and sharps containers. Needle-and-syringe services can be delivered by outreach workers in the community and can also be provided at various points of health service delivery – at visits to general practitioners, hospitals, OST sites, etc. Ideally, sufficient equipment should be provided to prevent needle and syringe re-use.† One-for-one exchange is not recommended because of an unintended increase in risk behaviour, because needle returns are influenced by a range of factors, including engagement with law enforcement. Mechanisms to support safe return of used equipment should be in place, as well as movements towards sharps boxes in community spaces.
Guidelines	Implementing comprehensive HIV and HCV programmes with people who inject drugs – practical guidance for collaborative interventions: https://www.inpud.net/sites/default/files/IDUIT 5Apr2017 for web.pdf Guide to starting and managing needle-and-syringe programmes: https://www.who.int/hiv/idu/OMSEA_NSP_Guide_100807.pdf

HCV, hepatitis C virus; TB, tuberculosis; OST, opioid substitution therapy; WHO, World Health Organization.

† , Guidance on starting a needle-and-syringe services is available at: https://apps.who.int/iris/bitstream/handle/10665/43816/9789241596275_eng.pdf?sequence=1.

BOX 12 Needle-and-syringe services in southern Africa.

By July 2020, needle-and-syringe services existed in nine South African health districts and in Maputo (Mozambique). They were also operational in Mauritius and several countries in East, West and Northern Africa.^5^

BOX 13 Behavioural interventions to support risk reduction.^19^

Interventions should be provided to help people who use drugs to support safer behaviours and sustain positive change. For people who use drugs, recommendations include the following:^19^

- involvement of people who use drugs in the development and delivery of messages
- interventions need to address risks related to drug use and sexual behaviour
- peer interventions are effective for the prevention and management of HIV and HCV
- information around safer injecting and drug use, as well as overdose prevention should be provided.

HCV, hepatitis C virus.

BOX 14 Low dead-space syringes.^73^

These syringes have 100-fold less blood in them compared with an ordinary syringe once the plunger has been pushed down fully. This reduces the survival of HIV and HCV within syringes. Where possible, these syringes should be provided, in consultation with needs and preferences of people who inject drugs.^73^

HCV, hepatitis C virus.

#### 2.4.2 Opioid substitution therapy

**TABLE 16:** Harm reduction interventions – Opioid substitution therapy.^74,81^

Variable	Description
Evidence and implementation experience	OST is the most effective treatment for opioid dependence.^74^ It is the practice of replacing an illegal opiate (such as heroin – also known as *nyaope* or *whoonga*) with a prescribed opioid agonist medication, such as methadone or buprenorphine (± naloxone), both of which are included in the WHO list of essential medicines. OST should be initiated and monitored by a clinician. OST is also known as medication-assisted treatment, opioid agonist therapy or, if methadone is used, as methadone maintenance therapy. OST is highly effective in reducing injecting drug use amongst opioid-dependent people, with reduced risks of HIV and HCV transmission. OST improves access and adherence to ART, reduces overdoses and associated mortality, lessens criminal activity and improves the physical and mental health of people with opioid dependency.^74^ In 2018, 86 countries were implementing OST.^5^ In South Africa, OST is provided as maintenance as part of out-patient services through private practitioners, through self-funded programmes at selected tertiary hospitals and through university- and civil-society-implemented programmes. OST (as maintenance) boasts higher retention in treatment and reduced use of un-prescribed opioids than managed withdrawal (detoxification); however, despite the sub-optimal outcomes of the latter, detoxification is still widely used.^75^ Clonidine and other non-opioid withdrawal management is sometimes used in practice, but it is not supported by evidence and should be avoided.
Main principles	Opioid dependence is a chronic condition, and ongoing recurrence of use is common. An assessment for opioid agonist/substitution therapy for maintenance should be performed in this context. The overall aim of OST is to treat opioid dependence through long-term provision of opioid agonist medications, at appropriate doses. It aims to improve health and well-being by reducing the risk of overdose, preventing cravings and withdrawal, reducing the health consequences of opioid use, and supporting social functioning. Counselling around and referral for voluntary psychosocial services will generally improve the outcomes of people on OST.^74^ OST with or without psychosocial support is useful. Clinicians should advocate for access to voluntary psychosocial support if it is needed, especially as it is under-valued and under-resourced by care providers and those providing resources, such as medical aids. The selection between methadone and buprenorphine (±naloxone) is a clinical decision that is made together with the patient after due consideration of: prior response, medical or mental health comorbidity, possible drug interactions, side-effect profile, cost/accessibility, use of other drugs and patient choice. Methadone, buprenorphine and buprenorphine-naloxone are registered for use for OST in South Africa. Efforts are underway to get these medications onto the EML for use at primary care level,† to lower cost and support clinicians around safe and effective OST service delivery. OST is most effective as a long-term maintenance treatment at appropriate dosages. OST should be provided for as long as required by the patient. A shorter duration of OST than needed is associated with higher rates of unregulated opioid use and increased risk for HIV and HCV transmission. Opioid withdrawal management (detoxification) should be avoided; it is associated with increased risks of death from overdose, when compared with providing no treatment, and with higher rates of return to use of opioids and increased risk of HIV and HCV transmission, when compared with long-term treatment.^76^ If detoxification is performed, then it should be tapered over at least 30 days, paired with intensive psychosocial services, and the person should be switched to OST if they are not able to be abstinent. Rapid tapering of opioid agonists (< 1 week) is contraindicated.^75^ Inpatient care for OST is required for a very small minority of patients; outpatient care is practical, safe and often successful and less costly.
Best practices	***Detailed clinical assessment at baseline/initial visit*:** A clinical assessment should consider the nature of substance use and related risks, complications, comorbidities, motivation to reduce use or quit and treatment goals. ***Clinical history:*** Basic demographics and a routine clinical history (current complaint, comorbidities and co-medication, prior illnesses, surgery, family history and allergies) are important, but attention needs to be paid to the items listed here. ***Clinical assessment:*** The clinical approach varies based on whether or not the patient is acutely ill. Generally, this includes assessment of various systems and general wellbeing, including a focus on signs suggestive of injecting, infectious disease (HIV, viral hepatitis, TB). An opioid withdrawal scale should be used to assess withdrawal when needed. ***Baseline testing:*** This should include HIV, HBsAg, HCV testing and, where relevant, selected testing (ALT, AST, FBC and creatinine) as clinically indicated. Patients who will receive methadone should have a baseline ECG, particularly those with cardiac risk factors or other medications that prolong QTc. Confirmation of opioid use, through urine or oral swab testing, is needed before starting OST.
Guidelines	Detailed guidance on OST is provided in Appendix 3. The National Department of Health is developing national guidelines for OST South African guidelines for the management of opioid use disorders: https://www.saams.co.za/Content/Documents/South_African_Guidelines_for_the_Management_of_Opioid_use_disorders_2015.pdf Guidelines for the psychosocially assisted pharmacological treatment of opioid dependence: https://www.who.int/substance_abuse/activities/treatment_opioid_dependence/en/

ALT, alanine transaminase; ART, antiretroviral therapy; AST, aspartate transaminase; ECG, electrocardiogram; EML, essential medicines list; FBC, full blood count; HBsAg, hepatitis B surface antigen; HCV, hepatitis C virus; OST, opioid substitution therapy; TB, tuberculosis; QTc, corrected QT interval; WHO, World Health Organization.

† , Methadone is included in the South African EML for opioid detoxification in hospital settings.

BOX 15 Testing for the presence of drugs in urine or other fluids.

Testing the presence of drugs in urine or other bodily fluids may be helpful to assist with diagnosis. Different views on the use of repeated drug testing exist. Testing is frequently used as a punitive measure and done in a way that violates the patient’s autonomy and dignity.^77^ The development of trusting therapeutic relationships and open discussions around concomitant drug use, based on the principles of harm reduction are recommended. However, some clinicians find drug tests useful as a clinical tool and to start discussions around concurrent drug use and on dose optimisation. If drug testing is done, it should be done with consent, and the results should be used in a supportive manner and kept confidential. Commercially available drug tests have significant false-positive and -negative rates and must be used with caution. False-positive urine tests for cannabis in patients using EFV (used in > 90% of HIV-positive South Africans at the time of writing this guideline) have been described.^78^

EFV, efavirenz.

BOX 16 Regulation of opioid substitution therapy medications and diversion.

Any doctor can prescribe OST. The quality of care and retention of patients can be improved through training. The scheduling of opiates means that prescribing is highly regulated, and close communication between the prescriber, the pharmacist and the person collecting the prescription requires active coordination. Diversion of OST medications at the patient level is an area of concern, but rarely a problem. However, the risk-benefit of take-home doses versus strict, continuous daily observed treatment are in favour of supported take-home doses. Diversion is usually an indication of limited access to OST. Patients on OST may share their medications with others as an act of solidarity and support.^79^ Tight OST medication procurement and stock control processes are important to minimise diversion.^80^

OST, opioid substitution therapy.

BOX 17 Stimulant drug use by people on opioid substitution therapy.^51^

People on OST may use stimulants because of OST-triggered fatigue, inability to experience pleasure or the desire to remain connected to the community of people who use drugs. Opioid substitution therapy is not designed to counter stimulant use, and the concurrent use of stimulants whilst on OST should not be viewed as a breach of treatment agreement and should not result in the reduction or discontinuation of OST. The benefits of OST are independent of stimulant use and limiting access to OST because of stimulant use denies the individual of the much-needed medication and is in contravention of the UNODC TreatNet principles.^77^

*Source:* UNODC.^51^

OST, opioid substitution therapy; UNODC, United Nations Office on Drugs and Crime.

BOX 18 Opioid substitution therapy and overdose risk.^84^

The risks of opioid overdose need to be known by patients and healthcare providers. Training on the use of naloxone is recommended, with take-home naloxone for all patients on OST. See section ‘Overdose services’ for additional details.

OST, opioid substitution therapy.

BOX 19 Opioid substitution therapy for special populations.^74,81^

Treatment is more complex in children and adolescents, pregnant or breastfeeding women, patients with significant medical comorbidity (e.g. hepatic impairment, HIV, TB and complex psychiatric pathology) and in patients with chronic pain who are dependent on prescription opioids. It is recommended that such patients be managed together with a specialist in the treatment of opioid use disorders.^81^

TB, tuberculosis.

BOX 20 Management of acute pain in opioid use disorders.

Patients with acute pain and opioid use disorders can be challenging to manage, partly because of central sensitisation, tolerance and opioid-induced hyperalgesia.^82^ Clinician-related barriers (including limited knowledge about opioid equivalent doses, stigmatisation and fear of overdose) may lead to poor analgesia in opioid-tolerant patients.

Note:^82^

- Pain is not controlled with methadone or buprenorphine for OST as it is dosed daily and the analgesic effect only lasts 4–8 h
- There is no evidence that exposure to opioid analgesics in the presence of acute pain increases rates of reuse of illicit opioids
- The additive effects of opioid analgesics and OST have not been shown to cause clinically significant respiratory or central nervous system depression
- Reports of acute pain with objective findings are unlikely to be manipulative gestures
- Additional guidance is provided in Appendix 3.

*Source*: Developed by Dr Urvisha Bhoora, a participant of the guideline workshop, for this guideline, drawing from the cited references

OST, opioid substitution therapy.

#### 2.4.3 Overdose services

**TABLE 17:** Harm reduction interventions – Overdose services.^83,84,85^

Variable	Description
Evidence and implementation experience	Globally, an estimated 167 750 deaths were directly associated with drug use in 2018; 76% of these were because of opioid use.^2^ Although overdose data in South Africa and the region are limited, UNODC estimates a drug overdose mortality rate of 12.5 per million persons aged 15–64 years in South Africa.^17^ Overdose from stimulant drugs also needs to be identified and managed. Figure 2 outlines the different approaches to take for managing sedative (e.g. from an opioid) or stimulant overdose.
**Main principles**	
Managing opioid overdose	**Identification** – the following opioid overdose triad should be observed:^83^ ■ Pinpoint pupils ■ Unconsciousness ■ Respiratory depression (bradypnoea) (< 10 breaths per minute or 1 breath every 5 s) ■ Patients may also have blue lips or fingernails, snoring/gasping and pale/clammy skin. **Response – SCARE ME** is an acronym of sequential steps in the event of suspected opioid overdose^84^ ■ S – stimulation (wakening) ■ C – call for medical help ■ A – airway ■ R – rescue breathing ■ E – evaluate breathing and response ■ M – muscular injection of naloxone ■ E – evaluate and support Standard resuscitation procedures should be carried out. Patients should then be considered for naloxone injection and referral for further management, if warranted.^84^ Using naloxone: Route of administration is based on the formulation available, skills in administration and the local context. Naloxone injection is available for SC, IM or IV injection or for IV infusion. Intramuscular naloxone may result in a more rapid clinical response. Intramuscular injection should be into a large muscle, preferably the deltoid or quadriceps muscle. In most cases 0.4 mg – 0.8 mg is an effective dose, with repeated doses as needed.^84^
2. Managing overdose because of benzodiazepines^85^	History of sedative use Presents drowsy, confused, pupils normal Check airway, breathing, circulation Provide respiratory support and give oxygen Administer flumazenil (0.2 mg IV over 15–30 s) ■ If no response after 30 s, then administer 0.3 mg over 30 s, 1 min later ■ If no response, then repeat dose of 0.5 mg IV over 30 s at 1-min intervals to a maximum cumulative dose of 3 mg/h ■ In the event of re-sedation, may repeat dose at 20-min intervals if needed; do not exceed 1 mg (administered as 0.5 mg/min) administered at any one time and no more than 3 mg/h ■ Rarely, patients may require titration up to a total dose of 5 mg: if no response after 5 min, then sedation is unlikely to be secondary to benzodiazepines Administer naloxone if opioid use cannot be excluded and observe response ■ Exclude other medical causes of confusion/sedation.
3. Managing stimulant overdose/intoxication^85,86^	**Identification** ■ History of stimulant use ■ Mild to moderate cases: dilated pupils, excited, racing thoughts, disordered thinking, strange behaviour, increased body temperature, flushed face, muscle cramps and stiffness ■ Severe cases: unresponsive, arrhythmias, circulatory and respiratory collapse, hypertension, tachycardia, fever, increased motor activity, seizure, confusion **Response** ■ Mild to moderate cases: observation, supportive management ■ Severe cases: ■ Treat with diazepam 5 mg – 10 mg PO, IV or PR until patient is lightly sedated ■ If psychotic symptoms do not respond, then consider anti-psychotic (e.g. haloperidol 1.0 g – 2.5 g PO or IM); refer to psychiatry as needed ■ In hospital: control seizures, treat cardiac complications, control blood pressure and temperature, secure IV access and correct any electrolyte imbalance
Guidelines	The WHO Mental Health Gap Action Programme (mhGAP) intervention guide for mental, neurological and substance use disorders in non-specialised health settings: https://apps.who.int/iris/bitstream/handle/10665/44406/9789241548069_eng.pdf?sequence=1 Community management of opioid overdose: https://apps.who.int/iris/bitstream/handle/10665/137462/9789241548816_eng.pdf;jsessionid=6FE0CF56D504F4C31D2B1E1F4E212FAF?sequence=1

IM, intramuscular; IV, intravenous; PO, *per os*; PR, per rectum; SC, subcutaneous; UNODC, United Nations Office on Drugs and Crime; WHO, World Health Organization.

**FIGURE 2:**
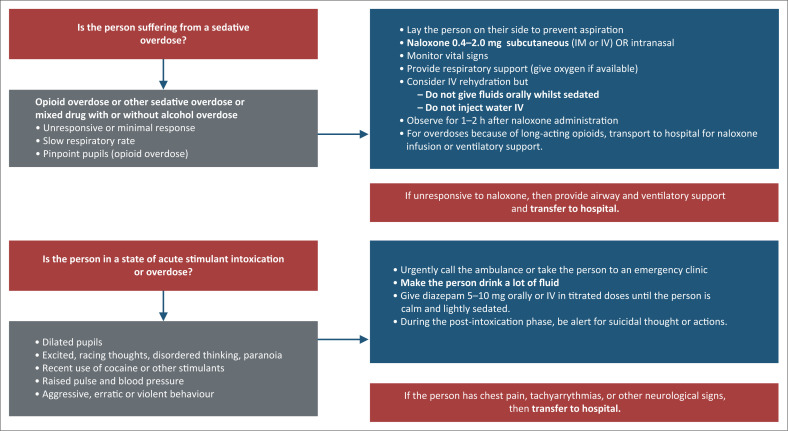
Algorithm for managing sedative or stimulant overdose.^19,87^

BOX 21 Opioid overdose prevention and reversal.^83,84,85^

Overdose can be both prevented and treated, thus staff in services working with people who inject drugs have an essential role in overdose prevention and management. Prevention interventions include:

- Education around overdose risks targeting people who use opioids
- Means to check the quality and purity of drugs using point of care drug testing kits by people who use drugs (see more in section ‘Emerging and ancillary interventions’)
- Drug consumption rooms/safe injecting facilities† (see more in Section ‘Drug consumption rooms’)
- Opioid substitution therapy services (see more in Appendix 3)
- Training around first responses and opioid overdose reversal using naloxone
- Expanding access to naloxone for overdose response:
  ■ Availability in community pharmacies, without a script
  ■ People likely to witness or respond to an overdose need to be trained in identification of overdose, naloxone administration and patient support and be provided with naloxone – these include ambulance, police and fire fighters, people who use opioids, people on OST and their family members or support people

OST, opioid substitution therapy.

†, It is noteworthy that no fatal overdoses have been reported in any of the countries, which implement safe injecting facilities.

BOX 22 Naloxone.^83,84,85^

This opiate antagonist reverses the effects of an opioid overdose, with a half-life of approximately 45 min. It is recommended by the WHO and is included in South Africa’s EML for use at primary level. The most distributed form is liquid naloxone for injection. It is also available as an auto-injectable or as a nasal spray (although these are not available locally). Like insulin for diabetes, naloxone can be administered safely by a lay person with very basic training.^84^ In South Africa, naloxone can be administered only by healthcare professionals.

EML, essential medicines list; WHO, World Health Organization.

BOX 23 Management of withdrawal for other substances.^88^

People experiencing withdrawal symptoms have developed dependence and should be referred for voluntary psychosocial services, and in some instances, medical treatment.

- Stimulants
  - Admission is usually not required
  - Beware of depression and assess suicide risk and psychosis
  - No substitution available; for severe symptoms provide 5–7 days of benzodiazepines
- Methaqualone
  - Withdrawal can lead to seizures or delirium; treat if symptomatic with diazepam (oral 5 mg 8-hourly, reducing over 3–5 days)
- Benzodiazepines
  - Avoid abrupt withdrawal; reduction takes time; patients require monitoring and motivation
  - Replace short-acting benzodiazepine with longer-acting benzodiazepine

#### 2.4.4 Emerging and ancillary interventions

Several additional interventions are important as part of comprehensive harm reduction. Some are briefly provided below, including (1) harm reduction for people who use stimulants, (2) drug consumption rooms and (3) drug checking services.

**2.4.4.1 Harm reduction for people who use stimulants:** Cocaine, methamphetamine (*tik, ice*), methcathinone (*cat*) and MDMA (*ecstasy*) are the most common unregulated stimulants seen in southern Africa, and methylphenidate is the most prescribed stimulant.

Simple harm reduction advice for people who use stimulants is to follow a few steps.

- Avoid the concurrent use of alcohol and cocaine. Cocaine use potentially compromises the cardiovascular system and is linked to several cardiovascular diseases; this risk increases with the concurrent use of alcohol.
- ***Rest:*** Sleep deprivation and stimulant use increase the chance of psychosis. People on stimulants often binge for days. People using stimulants should be encouraged to lie down in a dark space, with eyes close and relax for at least 3–4 h every 24 h.
- ***Hydrate:*** People using stimulants may be at risk of dehydration. People should be encouraged to drink 500 mL water per hour, especially if dancing.
- ***Eat:*** People using stimulants should be encouraged to eat something at least every 24 h, even if not hungry.
- ***Dental care:*** Sip water when the mouth is dry and brush teeth twice a day.

Other relevant harm reduction interventions include psychosocial support, condoms and lubricants (amphetamine-type stimulants can increase sex drive and risky sexual practice) and drug paraphernalia distribution (injecting and/or smoking kits that include mouth pieces for crack pipes), services for sexually transmitted infections, income generation and housing support. Substitution therapies for stimulant use disorders are under investigation.

**2.4.4.2 Drug consumption rooms:** Drug consumption rooms (also known as safe injecting facilities, medically supervised injecting sites or overdose prevention sites) are protected places for the hygienic consumption of drugs in a non-judgemental environment. They allow people to use drugs under medical supervision or in the presence of trained and equipped peers,^89^ enabling an immediate response to overdose and decreasing the transmission of blood-borne diseases through access to sterile injecting equipment and education on safe injection practices.^90^ Drug consumption rooms increase uptake of other health services and are an entry into care, for example, facilitating access to HIV, viral hepatitis, TB testing and treatment services and counselling.^90^ In 2018, 11 countries were operating drug consumption rooms across 117 sites.^5^ An overview of drug consumption rooms is available at: http://www.drugconsumptionroom-international.org.

BOX 24 Resources on harm reduction for stimulant use.

- Guidance for general practitioners working with people who use stimulants: https://www.rcgp.org.uk/-/media/Files/SMAH/RCGP-Guidance-for-working-with-cocaine-and-crack-users-in-primary-care-2004.ashx?la=en
- HIV prevention, treatment, care and support for people who use stimulant drugs: https://www.unodc.org/documents/hiv-aids/publications/People_who_use_drugs/19-04568_HIV_Prevention_Guide_ebook.pdf
- A community-based site with details about drug compositions and harm reduction: https://tripsit.me/about/

**2.4.4.3 Drug-checking services:** A means to check the quality and purity of drugs should be available to people who use drugs. This includes fixed site testing and on-the-spot testing options, the latter being mostly qualitative tests.^91^ For example, strips designed to identify fentanyl in drugs may help to prevent overdoses. People who use opioids can use the results of the test kit strip to inform their drug use (i.e. to use slowly, to reduce the volume of drug, to use in the company of others, to have a naloxone rescue kit nearby or not to use the substance). Test kits can be used on crushed pills or powders.^92^ Guidance around the use of fentanyl test strips is available at: https://harmreduction.org/issues/fentanyl.

#### Key points

- Harm reduction requires engaging with patients to identify immediate risks and develop means to reduce these.
- Screening, brief intervention and referral for treatment is an effective approach to detect and intervene in harmful substance use.
- Needle-and-syringe services are the cornerstone of HIV prevention for people who inject drugs and should be provided at all contacts with health services.
- People who inject drugs should be supported to return their used injecting equipment and locations for safe disposal of used equipment should be made widely available.
- Opioid substitution therapy is the most effective treatment for opioid use disorder. Effectiveness is maximised when patients are supported and provided with an optimal dose of medication. Voluntary psychosocial services can improve outcomes. Safety risks are greatest early on during treatment, and patients who are stable should be considered for take-home dosing. Treatment should be long term.
- Good supply chain and stock management are important to minimise diversion of opioid agonist medications.
- Opioid overdoses cause many deaths and are preventable. People likely to witness an opioid overdose should be trained to identify and respond to this, and access to naloxone should be maximised.

### 2.5 HIV testing, treatment and care

#### 2.5.1 HIV testing and counselling

**TABLE 18:** HIV testing treatment and care – HIV testing and counselling.^19^

Variable	Description
Evidence and implementation experience	People who use drugs are at risk of exposure to HIV and other STIs, owing to the drugs’ effects on the brain that may alter judgement and decision-making. The use of drugs, including alcohol, opioids, methamphetamine and other stimulants has demonstrated its influence on risky behaviours such as increased drive for, amongst others, sex, multiple sexual partners and unprotected sex.^19,51^ People who inject drugs are at higher risk of infection from HIV, HCV and other blood-borne diseases transmitted through the sharing of needles and syringes.^19^ HTS can be an entry point to HIV and viral hepatitis prevention and treatment services. Combining counselling with knowledge of a person’s HIV and HCV status can help link people to harm reduction services. *Vice versa*, offering HIV and viral hepatitis testing is part of harm reduction best practices.
Main principles	HTS may be provided in a variety of settings, including along outreach routes, in mobile units or temporary testing sites, at drop-in centres, needle-and-syringe services sites and dedicated HTS and health facilities. HTS services should also be provided to patients within detention settings as well as within prison. HTS can be either with finger-prick or oral swab samples. Oral swabbing may be more acceptable to people who inject drugs, in whom it may be difficult to access venous blood.^52^ Both the location and the timing of HTS should be responsive to the needs and requests of people who inject drugs. In some settings, this might mean providing services during evening hours or weekends, or at home through self-testing (described below).^52^ The provision of integrated services removes some of the perceived barriers to HIV testing for people who use drugs and is cost-saving. HIV services need to be especially ‘friendly’ to youth and women who use drugs. Social mobilisation and educational initiatives amongst networks of people who use drugs should be performed to encourage service uptake.
Approaches for systematic scale-up	**Methods of HTS delivery recommended for people who use drugs:** **HIV testing performed by peer outreach workers or lay providers:** HTS are often more acceptable to people who use drugs when performed by a trusted community member, that is, another person who uses drugs. Peer outreach workers can be an effective part of the HTS workforce. Adequate training, ongoing support and monitoring are essential. **HIV self-testing:** This is a process in which an individual performs a test and interprets the result by themselves, often in private. Self-testing does not provide a definitive diagnosis. A reactive (positive) self-test result requires confirmatory testing by a healthcare worker. Providers should demonstrate the use of the kit, which should contain easy-to-follow instructions and referral information for further assistance.^93^ The acceptability of HIV self-testing amongst people who use drugs has been shown in other regions.^94^ **Partner and family testing:** When a person tests HIV-positive, it is often helpful to offer voluntary testing of their sexual or injecting partners, spouses and family members. People who inject drugs living with HIV should be supported to disclose their results to their partners and trusted family members. **PICT:** Owing to the stigma and societal discrimination associated with drug use, people who use drugs are more likely to attend specific health services, such as needle-and-syringe programmes, drop-in centres, drug-dependence treatment and recovery services, OST programmes, mental health clinics or TB and STI clinics as specific to their needs.
Guidelines	South African HIV self-testing policy and guidance considerations: https://sahivsoc.org/Files/Self Testing_Guidelines_2017_WEB.pdf National HIV self-screening guidelines: https://www.aids.org.za/wp-content/uploads/2018/06/Final-HIVSS-guidelines-May-2018.pdf WHO recommendations for HIV testing by lay providers (2015): https://www.aidsdatahub.org/sites/default/files/publication/WHO_recommends_HIV_testing_by_lay_providers_2015.pdf.

HCV, hepatitis C virus; HTS, HIV testing services; STI, sexually transmitted infection; TB, tuberculosis; OST, opioid substitution therapy; PICT, provider-initiated counselling and testing.

#### 2.5.2 Antiretroviral therapy

**TABLE 19:** HIV testing treatment and care – antiretroviral therapy.

Variable	Description
Evidence and implementation experience	Despite stereotypes, with adequate social support and allied medical services, people who use drugs have levels of viral suppression that are similar to other groups of people.^95^
Main principles	People who use drugs and who are found to be HIV-positive should be offered immediate treatment or referral for long-term care and treatment, preferably at a clinic or hospital whose staff are respectful of everyone and diversity of choice. Community-based case management or peer navigation could facilitate treatment initiation and adherence support for people who use drugs. ART choices for common first-line regimens, including DTG are conventional and should follow national guidelines. As a result of potential drug–drug interactions, care should be taken when using the NNRTIs, especially EFV and NVP, as well as the NRTI, ABC, with methadone and buprenorphine; TDF, 3TC and RPV may be the safer drugs to use. PIs have many drug interactions with other medications, notably methadone, buprenorphine and psychiatric medications.
Guidelines	ART clinical guidelines for the management of HIV in adults, pregnancy, adolescents, children, infants and neonates (2019): https://sahivsoc.org/Files/2019 Abridged ART Guidelines 10 October 2019.pdf HIV drug interactions checker: https://www.hiv-druginteractions.org/

3TC, lamivudine; ABC, abacavir; ART, antiretroviral therapy; EFV, efavirenz; DTG, dolutegravir; NNRTIs, non-nucleoside reverse transcriptase inhibitors; NRTI, nucleoside reverse transcriptase inhibitor; NVP, nevirapine; PIs, Protease inhibitors; RPV, rilpivirine; TDF, tenofovir disoproxil fumarate.

#### 2.5.3 Prevention of mother-to-child transmission of HIV

**TABLE 20:** HIV testing treatment and care – prevention of mother-to-child transmission of HIV.^19^

Variable	Description
Evidence and implementation experience	PMTCT should involve: Primary prevention of HIV infection amongst women of child-bearing age Prevention of unintended pregnancies amongst women of child-bearing age living with HIV Prevention of HIV transmission from women living with HIV to their infants Provision of treatment, care and support to mothers living with HIV and their children and families
Main principles	PMTCT services should follow national guidelines. Women who use drugs experience additional barriers to accessing HIV services; special efforts are needed to understand and overcome these barriers. Additional information and guidance are provided in section ‘Women who use drugs’.
Guidelines	SAHCS Guidelines to support HIV-affected individuals and couples to achieve pregnancy safely: Update 2018, found here: https://doi.org/10.4102/sajhivmed.v21i1.1079 South African National Department of Health 2019 Guideline for the Prevention of Mother to Child Transmission of Communicable Infections (March 2020 update)

PMTCT, prevention of mother-to-child transmission of HIV; SAHCS, Southern African HIV Clinicians Society.

#### Key points

- People who use drugs should be informed of their rights to confidentiality and consent and their right to refuse HIV testing if they choose.
- Uptake and retention in care are improved where ART is integrated with OST when needed.
- Pregnant women living with HIV who are not on ART should be enrolled on an ART programme urgently.
- Needle-and-syringe programmes and other evidence-based harm reduction services should be offered to all people who use drugs, irrespective of their HIV status.
- All people who use drugs and who are found to be HIV-negative should be provided with risk reduction information and commodities tailored to their substance use (considering their patterns and type of substance, etc.) and sexual practices.
- People who use drugs and who test HIV-negative should test regularly (every 6 weeks to 3 months) depending on their risk profile.
- An individual with a discrepant HIV test result should be referred for re-testing in 14 days.
- If a person comes for HTS within 72 h after a potential exposure, then PEP should be considered^52^ (see section ‘Pre- and post-exposure prophylaxis’)

### 2.6 Prevention and management of coinfections and comorbidities

#### 2.6.1 Tuberculosis services

**TABLE 21:** Tuberculosis services.^98^

Variable	Description
Evidence and implementation experience	TB is frequently underdiagnosed amongst people who use drugs, particularly amongst people who use or inject heroin.^96,97^
Main principles	Chest X-rays should be considered in addition to TB symptom screening to rule out active TB amongst people who use drugs.^98^ In general, TB treatment and services are conventional. The propensity for TB drugs to have overlapping toxicity with other conditions with hepatic impact, such as HBV and HCV, and the medications used for treating these, means that careful counselling and follow-up should be available. Rifampicin can reduce the concentration and effect of heroin, methadone and buprenorphine, triggering opioid withdrawal and should be discussed as part of counselling. People who use drugs may have dysfunctional immune systems because of their drug use, and the inflammatory changes may mean that responses to TB treatment may be more unpredictable. Clinicians should pay close attention to these patients, especially with regards to any new symptoms. TB is often a marker of poor socio-economic status and, in the context of drug use, should trigger intensive and appropriate social, community and nutritional support. Ongoing drug use is not a contraindication to TB treatment. Clinicians should be aware that engaging with patients around ways to complete TB treatment and modify/adapt substance use patterns will likely result in higher treatment completion rates, compared with expectations of abstinence. Where possible, OST should be offered to people with opioid dependence and should be co-administered with TB treatment.
Guidelines	Integrating collaborative TB and HIV services within a comprehensive package of care for people who inject drugs: https://samumsf.org/sites/default/files/2018-10/WHO Care Package for people who inject drugs.pdf

TB, tuberculosis; HBV, hepatitis B virus; HCV, hepatitis C virus; OST, opioid substitution therapy.

#### 2.6.2 Viral hepatitis services

**TABLE 22:** Viral hepatitis services.

Variable	Description
Evidence and implementation experience	The risk of HCV infection amongst people who inject drugs is high where access to sterile injecting equipment and OST is limited. Regular HCV testing should be conducted in people who use drugs. Antibody testing is the first step for people of unknown status. Those who have resolved their infection or have been treated should receive follow-up testing for HCV RNA. Cure rates with DAAs – measured as sustainable virological response – amongst people who inject drugs are > 90% in most cases. All people who are infected with HCV are candidates for treatment.^99^
Main principles	Hepatitis prevention (including vaccination for HBV) testing and treatment should be integrated into services for people who use drugs. An alcohol intake assessment should be performed amongst patients with chronic HCV infection, linked to a brief intervention and further management as needed. Counselling around risk reduction to avoid HCV re-infection amongst people who inject drugs is important, as well as the provision/referral to access sterile injecting equipment and OST (for people who inject opioids). In HIV/HCV co-infected patients, potential interactions exist with ART medications for HIV treatment and DAAs for the treatment of HCV, sometime requiring altering of DAA dose. Screening for HBV infection should include assessment of HBsAg. Work-up and management for HBV infection is the same for all population groups. People with HBV-HCV co-infection should be managed by a specialist.
Approaches for systematic scale-up	HCV treatment services are restricted to academic treatment centres, and selected private providers, largely because of the cost of the drugs and relatively small numbers of patients. Advocacy efforts will be required to scale up these services and to reduce costs. HBV services should be integrated into existing services and uncomplicated cases should be managed at the primary care level.
Best practice/specific guidelines	National Department of Health clinical guidelines for the management of viral hepatitis: https://sahivsoc.org/SubHeader?slug=ndoh-and-who-guidelines University of Liverpool website and app for selected DAA therapy: https://www.hep-druginteractions.org Guidance on the prevention of HBV and HCV amongst people who use drugs: https://apps.who.int/iris/bitstream/handle/10665/75357/9789241504041_eng.pdf;jsessionid=0F5321E0AB93C580D133E92B666BC8CD?sequence=1 Guidelines for the care and treatment of persons diagnosed with chronic HCV infection: https://apps.who.int/iris/bitstream/handle/10665/273174/9789241550345-eng.pdf?ua=1 Guideline for the management of HIV/HBV co-infection: https://sahivsoc.org/Files/Guidelines on management of HIV hepatitis B co-infection (April 2011).pdf South African guideline for the management of chronic hepatitis B (2013): http://www.samj.org.za/index.php/samj/article/view/6452/5064

DAAs, direct-acting antivirals; HBV, hepatitis B virus; HCV, hepatitis C virus; RNA, ribonucleic acid; OST, opioid substitution therapy; ART, antiretroviral therapy.

#### 2.6.3 Mental health services

**TABLE 23:** Mental health services.

Variable	Description
Evidence and implementation experience	Common mental health disorders, including depression, anxiety and substance use disorders are twice as common in people living with HIV than in the general population and are also elevated amongst people with substance use disorders.^100^ Mental health challenges may be the result of psychosocial stress related to the diagnosis or other factors or may be directly attributed to neurological or other opportunistic infections or substances.
Main principles	Screening patients to identify harmful/risky substance use for mental disorders is crucial. Untreated substance use disorders and mental disorders, particularly amongst people living with HIV, may result in HIV treatment being less effective owing to compromised adherence and on-going risky behaviour.^101^ Presentation varies from physical complaints to behavioural disturbance such as social withdrawal, aggression or violence and may result in sub-optimal adherence and HIV disease progression. The availability of appropriate treatment and better known outcomes associated with early intervention means that these health problems can and should be addressed timeously to improve the likelihood of better outcomes in HIV treatment. Screening should always be carried out with due consideration of appropriate referral and follow-up. Addressing the potential risks associated with the interrelationship of drug use and HIV requires a multipronged, individualised approach.^57^ This means that social-inclusion-focussed psychosocial services are critical to deliver effective services for the prevention and treatment of HIV amongst people who use drugs. These include interventions such as MI, CM and CBT, based on the principles of individual and community inclusion and participation, peer support and the needs of the individual. Psychosocial interventions improve retention in ART compared with no intervention.^100^ Be aware of drug–drug interactions between psychiatric medications and OST. Where referral for additional assessment is warranted, this is carried out in accordance with the *South African Mental Health Care Act* No. 17 of 2002.^102^
Guidelines and resources	Mental health information centre of southern Africa: https://mentalhealthsa.org.za/ South African Depression and Anxiety group: http://www.sadag.org/ Materials for the provision of psycho-education for mental disorders via the Mental Health Innovation Network: https://www.mhinnovation.net/resources mhGAP intervention guide: https://www.who.int/publications-detail/mhgap-intervention-guide---version-2.0

ART, antiretroviral therapy; CBT, cognitive behavioural therapy; CM, contingency management; MI, motivational interviewing; OST, opioid substitution therapy.

#### 2.6.4 Sexual and reproductive health services

**TABLE 24:** Sexual and reproductive health services.

Variable	Description
Evidence and implementation experience	The SRH of people who use drugs is often overlooked by healthcare providers. All people should be able to enjoy pleasurable sexual lives and have equal access to SRH services and rights.
Main principles	Screening, diagnosis and treatment of STIs should be provided routinely. Clinicians should be able to have open and honest discussions with people who use drugs, around sexual practices and risks, including high-risk practices (e.g. unprotected anal or vaginal intercourse and multiple partnerships) and around sex work. Contraceptive services should be offered to women who use drugs. Women who use opioids should be informed about the influence of opioids on menstruation and the potential for pregnancy in the absence of contraception. There are no clinically significant drug interactions with opioid agonist medications and hormonal contraceptives. Dual contraception methods should be advised to women at high risk for HIV. Clinicians should provide termination of pregnancy services aligned with local guidelines. Women who use drugs may not actively engage in healthcare services, so cervical cancer screening should be integrated into harm reduction service delivery. Support and care should be provided to women who use drugs during conception and care.
Guidelines and resources	UNODC guidelines on drug prevention and treatment for girls and women (2016): https://www.unodc.org/documents/drug-prevention-and-treatment/unodc_2016_drug_prevention_and_treatment_for_girls_and_women_E.pdf WHO guidelines for the identification and management of substance use and substance use disorders in pregnancy (2014): https://apps.who.int/iris/bitstream/handle/10665/107130/9789241548731_eng.pdf?sequence=1

SRH, sexual and reproductive health; STIs, sexually transmitted infections; UNODC, United Nations Office on Drugs and Crime; WHO, World Health Organization.

### 2.7 Critical enablers

#### 2.7.1 Supportive law and policy

Clinicians, programme managers and policymakers should work together to support the decriminalisation of drug use, as well as sex work, to reduce health risks related to arrest, detention and incarceration and ensure the protection of rights.

Interventions and support to reduce drug dependence should ideally consist of a continuum of care, starting with early development strategies focusing on the delay of drug use and prevention strategies, moving to early-use interventions such as brief interventions and information. More intensive interventions should be reserved for people with dependencies that cause significant impairment.^85^ A supportive and effective continuum and continuity of care service requires supportive policies. The criminalisation of people who use drugs often disrupts the provision of a continuum of care, by seeing all drug use as a criminal act, thus disrupting the continuity of services through arrest and incarceration and accelerating the development of drug dependence.^103,104^ Considering the additional economic, health, social and psychological harms associated with the criminalisation, arrest and incarceration of people who use drugs, there should be a robust debate on the decriminalisation of the use of drugs and advocacy for the provision of services for incarcerated populations. The Southern African HIV Clinicians Society supports the decriminalisation of drug use: https://sahivsoc.org/Files/2019-06-03%20Drug%20Use%20%20Decrim%20statment.pdf

Clinicians should take on an advocacy role for: better care based on evidence for people who use drugs; human rights for people who use drugs; harm reduction approaches that include the activities described earlier, as well as access a safe supply of opioids (see the text Box 26 on safe supply). Clinicians should also advocate for the evaluation of current policies regarding illicit drug law and enforcement.

#### 2.7.2 Countering stigma and discrimination

Clinicians and public health leaders should work with civil society organisations and networks of people who use drugs to monitor stigma and discrimination and advocate to change punitive legal and social norms. The development of a stigma index that includes people who use drugs and other people engaged in illegal and/or stigmatised practices could be developed to quantify stigma and measure changes over time.

BOX 25 Decriminalisation.

Several nations are experimenting with decriminalisation of drug use or possession for personal use (e.g. Portugal) with significant improvements in public health, particularly around HIV and other health conditions.^105^

BOX 26 Safe supply interventions.

These interventions aim to address the harms related to contaminated drug supply and remove the risks associated with unknown strength of substances and potential toxicity of additional substances. These interventions provide alternatives to street-level drugs. Such programmes exist in Canada and include the prescription by doctors of hydromorphone and diacetylmorphine for people who use opioids.^106^ Safe supply requires policy changes that decriminalise drug use and support the prescription of drugs.

Clinicians should ensure that the health services they provide are available, accessible and acceptable to people who use drugs.

Approaches to rendering services friendly to people who use drugs and other key populations:

- Ensuring adequate training of staff and develop supportive attitudes towards people who use drugs
- Integrating health services
- Providing services at times that suit patients
- Locating services strategically where patients congregate or transit
- Involving peers in the planning, promotion, delivery and monitoring of services
- Taking steps to ensure law enforcement does not interfere with access to services

#### 2.7.3 Enabling community empowerment

Clinicians, public health leaders and civil society organisations can support the empowerment of people who use drugs by enabling their active participation in the planning and implementation of services, with a focus on peer education and training on safer drug use, harm reduction and broader issues relating to their rights and health.

#### 2.7.4 Acting against violence

People who use drugs are at high risk for physical, sexual and psychological violence. This violence increases their risk for HIV and viral hepatitis and negatively affects their mental health. Many people who use drugs have been traumatised through their engagement with law enforcement and entry into the criminal justice system. Women who use drugs are at particularly high risk of violence and its effects.

Clinicians, public health leaders and civil society organisations should aim to prevent violence affecting people who use drugs, which can include engagement with law enforcement to sensitise them to the issues and their role to uphold the rights of all people.

The occurrence of violence should also be monitored and reported and mechanisms to access redress explored.

Clinicians should provide clinical care and initial psychological support to survivors of violence, with referral for additional support when needed. Processes following instances of rape should follow local guidelines.

There is strong evidence linking structural inequities to accessing health services with a higher risk of HIV infection, as well as continuing or everyday intimate partner- and gender-based violence.^107,108^ Structural inequities in access to services hold true particularly for people who use licit drugs in countries where drug use has been criminalised and where no harm reduction services exist. To address the concerns emerging from the many interacting aspects of violence, trauma and substance use, harm-reducing systems of care need to integrate with other primary healthcare services.^109^ Linking harm reduction services to services such as sexual and reproductive health (SRH) services, including sexually transmitted infection (STI) prevention services, and supportive primary care would allow for more effective harm reduction programming.

## 3. Special considerations

### 3.1 Young people who use drugs

**TABLE 25:** Young people who use drugs.

Variable	Description
Important issues	Young people experience barriers to accessing harm reduction services when they are aged <18 years because of several factors, including staff attitudes, organisational policies and practices and laws.^110^ Youth-specific harm reduction services are rare,^110^ leaving a gap between age of initiation into drug use and the age at which services are accessible.^111^ Adolescents who inject drugs differ from their older counterparts in terms of socio-economic factors, risk behaviours and the kinds of drugs consumed. Increased injecting risks occur amongst specific subgroups including young street dwellers, girls, ethnic minorities, survivors of sexual abuse and those with low educational attainment or who are out of school.
Main principles	The Commission on the Rights of the Child suggests: ■ Non-criminalisation, which mandates non-compliance of healthcare providers with arrest-based interventions, an immediate end to arrest and prosecution of adolescent key populations aged 10–17 years and the abolition of involuntary custodial placement in the name of ‘rehabilitation’. ■ Voluntary, confidential and adolescent friendly primary SRH services ■ Respecting the right of adolescents aged 10–17 years who sell sex or use drugs to be heard, including meaningful participation in policy and decision-making in health services and other programmes that concern them, as well as reliable complaint procedures and remedies for rights violations. ■ Waiving the need for parental consent for life-saving SRH, HIV and harm reduction services. ■ Obtain patient-centred informed consent and respect for the right to refuse or consent to medical treatment and participate in research trials. ■ Tailor harm reduction services to the age, gender and risk profiles of recipients. Young people who engage in sex work and those who engage in same-sex practices require further tailored services. ■ Implement targeted comprehensive services for young people who use drugs to include interventions integrated into the already existing HIV prevention and care programmes including school friendly opening times to access these services. ■ Social protection services are an especially important part of harm reduction services for young people. Ensuring access to cash plus care programmes for adolescents from difficult financial and social circumstances renders them less vulnerable and can assist in retention-in-care programmes^112^ and may have a role in OST. ■ Include accessible and practical information about HIV and AIDS and TB, mental health, SRH, substance use and harm reduction as part of the school curriculum.^111^ Interactive behavioural skills practice (such as role-plays) and non-judgemental, non-moralising forms of engagement and education are vital for effective and inclusive health communication with youth.^111^ ■ Work with mobile-health innovations to create application-based programmes that promote engagement of youth with services. This could, for instance, follow the concept of the ‘Happy Hour’ programme implemented in KwaZulu-Natal.^112^
Guidelines	National adolescent and youth health policy (2017): http://www.health.gov.za/index.php/shortcodes/2015-03-29-10-42-47/2015-04-30-08-18-10/2015-04-30-08-25-54?download=2459:adolescent-and-youth-policy-4-sept-2017 Gazetted, comprehensive information on drug testing in schools: http://www.education.gov.za/Portals/0/Documents/Publications/Drug%20Testing%20Guide_FINAL_PRINT.pdf?ver=2014-07-18-150102-000

AIDS, acquired immune deficiency syndrome; OST; opioid substitution therapy; SRH, sexual and reproductive health; TB tuberculosis.

BOX 27 Drug searches and testing in schools.^113^

Random search and seizure and drug testing are routinely practised in schools as part of efforts intended to safeguard the right to education in environments free of drugs and dangerous objects.

- Searches can take place if there is suspicion of use by a learner†
- Searches can only be conducted by trained educators.
- Drug testing must be conducted by the principal or delegated to someone of the same gender as the learner.
- Testing must be done in private and the process and results managed confidentially.
- The goal is to get support for the learner to stop using drugs and get back into school in a non-punitive and respectful manner.
- Counselling must be done by social workers and NGOs. No criminal proceedings for drug use may be brought against the learner.
- Selling drugs is more serious and suspension, expulsion and involvement of the police may be indicated.

NGOs, non-governmental organisations.

†, Suspicion of use may arise from students informing the principal about the presence of drugs on school premises, scent of drugs, report by parents, traces of drugs on premises and other reasonable indications.

### 3.2 Women who use drugs

**TABLE 26:** Women who use drugs.

Variable	Description
Important issues	In southern Africa, a large proportion of women who use drugs are homeless, unemployed and inject illicit drugs. Many engage in high-risk behaviours – such as sex work – in order to survive. This places them at risk of contracting HIV, viral hepatitis and STIs and to face stigma and discrimination from healthcare workers.^115^ Women report feeling uncomfortable when accessing services when they feel outnumbered by the males who use drugs.^115^
Considerations	Recommendations for gender-sensitive programmes:^19,114,115,116,117^ Create safe spaces for women: ■ Harm reduction services should cater for the needs of women with due consideration of multiple and diverse identities. ■ Special attention should be paid to the location of services, operating hours and availability of childcare. ■ As women are at high risk of intimate partner violence, they should be given the option to be treated as an individual rather than as a couple and in a space where they are protected from persons who may have assaulted them. ■ Women who report abuse should be referred for social support services. ■ Care should be taken to avoid re-traumatising women in mixed treatment groups. Offer women comprehensive prevention services: ■ Women who use drugs should be screened routinely for HIV, TB, viral hepatitis, STIs and cervical cancer. ■ If HIV-negative, then offer PrEP. ■ If HBV-negative, then offer HBV vaccination. ■ Sufficient quantities of condoms with condom-compatible lubricants should be offered to all women. ■ Screening for mental health conditions, such as depression and psychosocial stress, should occur on a regular basis, with referral to **psychosocial services**, especially to deal with instances of physical and emotional violence and abuse. ■ Services should establish support groups and provide information and guidance on reporting and receiving assistance for human rights violations. ■ SRH and rights must be considered, including access to safe and effective contraception, as well as non-judgemental and comprehensive treatment of STIs. ■ Pregnant women who use drugs should be referred to high-risk antenatal care services when needed. Methadone and buprenorphine improve outcomes for opioid-dependent mothers and their infants. Withdrawal during pregnancy should be discouraged because of adverse pregnancy outcomes and OST should be offered. ■ Non-evidence-based practices such as insisting on abstinence during pregnancy; withholding pain medication during labour; separating infants from mothers immediately after birth; coerced or forced sterilisation, should actively be rejected. Women who use drugs should also be assisted with accessing emergency contraception and termination of pregnancy services if so desired. Provide comprehensive treatment: ■ Women who use drugs should be provided with comprehensive treatment, especially for HIV, TB, HBV, HCV and mental health conditions, ideally in one visit and with sensitive and appropriate adherence support. The principles of ART, as outlined in the Southern African HIV Clinicians Society guidelines should be followed. Newer regimens incorporating InSTI-based ART are preferred and should be used when available. Healthcare workers should be cognisant of, and sympathetic towards the lived realities of women who use drugs where theft and confiscation of belongings, including ART, are common occurrences. ■ Women who use drugs should be counselled accurately around the teratogenicity of alcohol and drugs. ■ ‘Potential drug interactions should be avoided’, especially when co-prescribing HIV and TB treatment, contraception, HCV, OST and psychiatric medications ■ Programmes should sensitise and train healthcare workers in appropriate emergency care. Women who use drugs are less likely to receive timely management of overdose because of a low level of suspicion and awareness amongst the public and healthcare workers.^118^ ■ Women who use drugs should be trained to recognise suspected opioid overdose and should ideally have access to naloxone and be instructed in its use for emergency situations. ■ Healthcare services should be mobilised and combined with existing harm reduction facilities that have connections with women who use drugs.
Guidelines	Advancing the SRH and rights of women who use drugs – a guide for programmes: https://frontlineaids.org/wp-content/uploads/2020/02/Guide-for-harm-reduction-programmes-FINAL-24Feb-WEB.pdf. UNODC guidelines on drug prevention and treatment for girls and women (2016): https://www.unodc.org/documents/drug-prevention-and-treatment/unodc_2016_drug_prevention_and_treatment_for_girls_and_women_E.pdf WHO guidelines for the identification and management of substance use and substance use disorders in pregnancy (2014): https://apps.who.int/iris/bitstream/handle/10665/107130/9789241548731_eng.pdf?sequence=1

*Source*: Auerbach and Smith^114^

ART, antiretroviral therapy; HBV, hepatitis B virus; HCV, hepatitis C virus; OST, opioid substitution therapy; PrEP, pre-exposure prophylaxis; SRH, sexual and reproductive health; STIs, sexually transmitted infections; TB, tuberculosis; UNODC, United Nations Office on Drugs and Crime; WHO, World Health Organization.

BOX 28 Gender-sensitive responses to drug use.^114^

Gender bias not only undermines women’s human rights and dignity but also leads to the development of harm reduction programmes, which do not accommodate their special needs. Programmes rarely consider women’s multiple roles, identities and socio-economic realities or the importance of the possible synergistic effects of age, class, gender, race/ethnicity and nationality with other identities.^114^

BOX 29 Transgender people.^120^

‘Transgender (trans) people are individuals whose gender identity or gender expression differs from what is typically associated with the sex they were assigned at birth. Many trans people are prescribed hormones by their doctors to change their bodies to reflect their gender identity. Some undergo surgery, but not all trans people can or will take those steps’.^119^ For transgender individuals, rates and risk factors for substance use, mental health problems and HIV are considerably higher than those of cis-gender counterparts.

- **Gender affirmation:** When caring for a transgender person, enquire about their preferred name and pronoun (he or him, she or her, they or them) and address them according to their preference.
- **Injections and needle-sharing**: Amongst transgender women, soft tissue fillers are commonly injected in the face, breast and hips and/or buttocks to feminise the contour of the face and body. Transgender men may inject testosterone for masculinisation, and transgender women may inject oestrogen (although use of oral Estrofem, Premarin and off-label contraceptives is more common than injections). Needle-and-syringe programmes are important for transgender people who require sterile injecting equipment to safely inject hormones and other substances for gender affirmation.^120^
- **Opioids and OST:** The use of opioids and OST has been linked to low testosterone level,^120^ which may have an impact on gender transitioning of transgender men. Monitoring of testosterone levels and adjustment of hormone replacement therapy dose may be necessary for transgender men using opioids or OST.

OST, opioid substitution therapy.

### 3.3 Substance use in the context of sexual encounters

**TABLE 27:** Substance use in the context of sexual encounters.

Variable	Description
Important issues	Drugs may be used in a range of sexual encounters, largely to enhance sexual pleasure. These include within heterosexual and same-sex relationships, as well in the context of sex work. A range of drugs are used. In the context of Chemsex,† which is usually seen amongst MSM, common drugs used include GHB, GBL, methamphetamine, mephedrone and related substances, used in a variety of ways (orally, inhaled, injected, per rectum). Chemsex may occur among couples or groups of people in a range of settings. Sexual encounters may last up to several days. Chemsex is associated with a high risk of STIs^120^; HIV transmission can be reduced through effective use of PrEP and maintaining HIV viral suppression amongst people living with HIV.
Considerations	The risk of overdose is highest related to GHB, which can cause cardio-respiratory depression and death. Physiological dependence on GHB may occur rapidly, within several days of repeated use within 24 h, and withdrawal may result in delirium tremens. Patients should be aware of risks for sexually transmitted infections, with discussions around PrEP and U=U. Counselling should be provided around choices, including condoms, lubrication, sero-sorting and sero-positioning. Support should be provided around engaging around issues relating to consent. Neutral, non-judgemental attitudes are required when counselling around these practices.
Resources and guidelines	Chemsex first aid: https://menrus.co.uk/wp-content/uploads/2019/01/Chemsex-First-Aid-action-sheet.pdf Tripsit – resource of novel psychoactive substances and harm reduction: https://tripsit.me/

GHB, gamma-hydroxybutyric acid; GBL, gamma-butyrolactone; MSM, men who have sex with men; PrEP, pre-exposure prophylaxis; STI, sexually transmitted infection; U=U, undetectable = untransmittable.

† , Chemsex, or sexualised drug use, is the intentional use of substances before or during sex to facilitate or to enhance sexual practices and pleasure, particularly amongst MSM.^121^

### 3.4 Prison settings

**TABLE 28:** Prison settings.

Variable	Description
Important issues	Some 56% – 90% of people who inject drugs will be incarcerated at some stage in their lives^122^ and drug use, including injecting, in the prison setting is widely documented. The prevalence of HIV, TB and HCV is higher in prison populations than in the general population. The failure of countries to implement comprehensive harm reduction measures in places of detention – including needle-and-syringe services and OST – violates their obligations under international human rights law. The effective diagnosis and management of HIV, viral hepatitis and TB in prisons benefits society as a whole as most prisoners return to the community. Good prison health is therefore public health. TB and HIV testing and treatment for inmates in correctional facilities in southern Africa has improved. Screening for substance use, mental health conditions and viral hepatitis is not done routinely. Few prisons employ evidence-based interventions around substance use and, with the exception of Mauritius, OST is not provided. No correctional services facilities in the region have needle-and-syringe services. Recidivism is between 70% and 98% amongst substance-using persons imprisoned for drug-related crimes and not treated during the period of their incarceration.
Considerations	Comprehensive screening and testing (including HIV, HBV, HCV and TB) should be performed at admission, biannually for sentenced offenders and on release.^19^ Inmates need to be informed fully and should provide consent to substance use and medical-related testing and treatment. This presents opportunities to incorporate harm reduction service as part of routine interventions. It is recommended to screen all inmates for problematic substance use in a non-judgemental, confidential manner using a validated screening tool. For those with opioid dependence, OST should be offered upon entry, during prison stay and continuity of OST upon release – linked to community support services. Pre-release interventions should ensure that inmates participate in overdose prevention awareness programmes.^83^
Resources and guidelines	Guidelines for the management of TB, HIV and STIs in correctional facilities: https://www.health-e.org.za/2014/06/12/guidelines-management-tb-hiv-stis-correctional-facilities/ Handbook for starting and managing needle-and-syringe programmes in prisons and other closed settings: https://www.unodc.org/documents/hiv-aids/publications/Prisons_and_other_closed_settings/ADV_COPY_NSP_PRISON_AUG_2014.pdf

HBV, hepatitis B virus; HCV, hepatitis C virus; OST, opioid substitution therapy; TB, tuberculosis; STI, sexually transmitted infection.

## 4. Recommendations

This section summarises the key role of critical stakeholders in the delivery of harm reduction services in HIV, TB, viral hepatitis and related services.

**TABLE 29:** Summary of harm reduction recommendations per provider/stakeholder.

Variable	Description
Pharmacists	Community pharmacies should increase support for needle-and-syringe services Provide a non-stigmatising service and ensure people who use drugs are not prevented from purchasing equipment Provide sharps disposal bins Offer referrals to access other harm reduction services Explore options to support directly observed therapy for OST services
Law enforcement and police	Develop linkages with social, health and harm reduction services, for example, see the Law Enforcement Assisted Diversion: https://www.leadbureau.org/ Ensure policies and processes are in place for continuation of chronic medication amongst people who use drugs, moving towards access to opioid agonists to avoid withdrawal whilst in custody
Emergency services	Ensure staff are sensitised to the needs and issues affecting people who use drugs Ensure staff are trained to identify and manage overdose Ensure all vehicles are stocked with naloxone Ensure staff are sensitised around supportive service provision for people who use drugs and drug-related emergency management protocols
Social service providers	The Southern African Society of Social Workers should become familiar with evidence and approaches supporting harm reduction and recommend inclusion of evidence-based interventions and services around substance use, particularly for people who inject drugs and those with opioid use disorders.
Programme managers	Ensure managers are familiar with the key harm reduction interventions Ensure programme managers know practical tips on how to coordinate harm reduction programmes
Policymakers	Ensure policymakers understand the importance of harm reduction as an essential component in addressing substance-use-related harms.
Higher education and health professional training	Ensure curricula cover drug use, human rights and evidence-based interventions relating to drug use
Communities	Ensure communities have the right information on harm reduction Schools should provide scientifically accurate information around substances in an honest and interactive manner that is of use to learners. The risks and benefits of drug testing should be assessed and be compassionate.^123^
Civil society	Ensure civil society is conversant with harm reduction principles, with a focus on practical application
Funders	Ensure that funds for HIV and viral hepatitis services can be used to cover the full package of comprehensive services recommended by the WHO, including the purchasing of needles and syringes Support and fund screening, brief interventions and referrals for substance use and mental health as part of HIV prevention and treatment programmes across populations and settings
Schools	Provide accurate information around drug use and treatment options as part of the school curriculum Be aware of the negative consequences of inappropriate management of substance use amongst learners Link with supportive networks of social and health providers around managing substance use amongst learners
Medical aids	Cover the costs of packages of services for people who use drugs Support OST maintenance therapy for people with opioid use disorder
Employers	Section 8 of the *Occupational Health and Safety Act* (OHSA) requires every employer to provide and maintain a working environment that is safe and without risk to the health of its employees. The General Safety Regulations made in terms of the OHSA state that an employer may not permit any person who is or who appears to be under the influence of intoxicating liquor or drugs to enter or remain at the workplace. An employee under the influence of a substance must be excluded from the workplace for safety reasons if there is a danger to themselves or others by the virtue of the nature of their work. Many people who use drugs remain highly functional and a blanket ‘zero tolerance’ approach towards drug use does not appreciate the fact that drug use is a chronic issue, with the likelihood of resolution of harmful use taking place in the context of a supportive environment. Many employers have a ‘zero tolerance’ approach not only to being under the influence of drugs and alcohol but also to testing positive to such substances. One must distinguish between ‘being under the influence’ and ‘testing positive’ – if an employee tests positive for a substance, this does not mean that they are under the influence: ‘Being under the influence’ means that the drug or alcohol has impaired the employee’s faculties to the extent that the employee is unable to function in their position as required and comply with the company’s policies and procedures. ‘Testing positive’ for a drug or alcohol means that following a breathalyser, urine or blood test, the employee has tested positive for that substance. A ‘zero tolerance’ approach can also extend to not permitting an employee to be in possession of a substance or use a substance at work. The General Safety Regulations made in terms of the OHSA make an exception for medicines at work, but stipulate that an employee is not allowed to work if the side-effects of the medicine constitute a threat to the health and safety of the employee or others. A ‘zero tolerance’ policy also should not necessarily always imply that any breach will result in dismissal. Whether a breach is dismissible should depend on a variety of factors, including the employer’s policies, the employee’s circumstances, the nature of the job and the circumstances of the infringement. Employers often insist in having a clause in the contract of employment providing that the employee will reasonably submit to drug testing, alternatively the company has a comprehensive occupational health policy in place providing for this. However, Section 7 of the Employment Equity Act allows testing, providing that: ‘medical testing of an employee is prohibited, unless – (1) legislation permits or requires the testing; or (2) it is justifiable in the light of medical facts, employment conditions, social policy, the fair distribution of employee benefits or the inherent requirements of a job’. The reference to ‘employment conditions’ could include factors as the impact of an intoxicated employee in the workplace. As drug testing is unreliable and gives false-positives and ‘does not necessarily correlate to the level of impairment’, meaning that ‘the presence of a positive drug test does not necessarily confirm that the worker was impaired at the time of the work-related incident or accident’.^124^ It is recommended that employers employ a system of testing for functional impairment rather than the presence of drugs.^124^

OHSA, Occupational Health and Safety Act; OST, opioid substitution therapy; WHO, World Health Organization.

BOX 30 Recommendations for employees who are hiring people who use drugs.^124^

1. **Pay attention to recruitment** not only for peers but also for the staff managing peers. It is a good practice to discuss in advance with the team, involving peers and managers, about the desired profile and skills of new staff. Involve peers in all steps of the recruitment process.
2. **Offer diverse work-engagement levels** like part-time, *ad hoc* activities or volunteering. Not everyone will be ready or willing to work full-time or in specific outreach functions. Offering different levels of engagement with work creates opportunities for people who use drugs to progress through the organisation whilst respecting their possibilities and needs at a given moment.
3. **Promote a harm reduction approach to drug use of staff**. Develop non-prohibitionist regulations at the workplace and focus on job performance instead of on drug use. What matters is that staff needs to be fit for work and must protect the organisation’s image. They must be accountable for their performance, regardless of their eventual drug use.
4. **Foster a supportive work environment**. Be appreciative and promote trust-building. Provide good work conditions and support workers’ needs and self-care. Be flexible with working hours when staff needs to attend OST or HIV/HCV/other treatment. Also, be understanding of performance problems caused by side-effects of medication, for instance.
5. **Build and sustain boundaries**. This implies not only being transparent about rules and how they are applied for everyone but also help to recognise, build and sustain boundaries to help protect staff from emotional burden.
6. **Promote diversity and respect within the team**. Invest in team care: ensure excellent communication, team-building and promote an environment of trust amongst colleagues. Foster the building of a diverse team and promote respect for this diversity within the team and the organisation.
7. **Promote meaningful involvement of staff members who use drugs** at all levels, not only on service delivery. Include staff in planning, evaluating and policy decision making. This may mean helping to prepare staff on how to give feedback, as some might have internalised stigma, which can create additional difficulties in sharing ideas.

OST, opioid substitution therapy; HCV, hepatitis C virus.

## Appendix 1: Common myths about drugs use

**TABLE 1-A1:** Common myths about drug use.†

Myth	Truth	Discussion	Implications
Once an ‘addict’, always an ‘addict’^126,127^	Not all people who use drugs develop drug-use problems. Population studies show that most people who develop a substance use disorder will resolve their drug issues and habituated use, most without ‘addiction treatment’.	Most people with a substance use disorder will resolve the disorder, with or without treatment. Most people resolve their substance use disorder as they mature and take on other interests and responsibilities, such as employment, marriage and raising a family. Most people in the United States resolve their substance use disorder by the age of 30 years. Divorce, unemployment, contact with the criminal justice system, stigma and exclusion can increase the duration of a substance use disorder.	The clinician’s role should be to limit the potential health consequences that are likely to outlive the problematic use of substances and reduce the risk of death before, during or after the substance use disorder has been resolved.
To be dependent is to be ‘addicted’^128^	Dependence is a normal state of physiology, whilst ‘addiction’‡ is a state of mind.	People become dependent on several medications. This means that their body will have an adverse reaction if they stop the medication. It does not talk to patterns of use or the negative consequences of the drug – most people dependent on drugs have positive outcomes: for instance, hypertensive medications, insulin and methylphenidate are all medications people use dependently and usually with no additional drug-seeking, excess dosing and purchasing from the illicit economy. Another example is coffee – many people need their first cup of coffee but are not addicted to it. ‘Addiction’ is when the drug takes on undue salience and meaning – the person actually disrupts their life and responsibilities to ensure that they get the drug, and the patterns of use are erratic, compulsive and they take higher doses than are beneficial. Simply put, the drug becomes one of their primary relationships. People often refer to OST medications as ‘addictive’. Whilst it can be ‘addictive’ to a small minority of people, many will be dependent on it and enjoy significant benefits from the medication without causing a disruption in their life and find that they are able to stabilise their situation.	Maintenance prescribing should not be time-limited, and dependence on the medication is no indication of impaired functioning.^9^
Abstinence is essential before being able to assist people in other areas of their life, and is essential for the resolution of substance use disorders^124^	The use of drugs is fluid and can be both beneficial and problematic. Abstinence from certain drugs may be beneficial or detrimental to the individual. Most studies have shown that people who moderate or down-titrate to abstinence do as well, if not better than those who aim for immediate abstinence. Change is a process and drug-related problems are often only resolved when other problems are resolved.	There is limited literature on the comparative outcomes of controlled/reduced use compared with abstinence for substances other than alcohol. Studies comparing outcomes of controlled or reduced use versus abstinence from alcohol confirm that abstinence is the safest goal, but evidence supports the value of reduced drinking approaches as part of the spectrum of interventions for alcohol use disorder.	Abstinence should not be a criterion for determining functionality or someone’s character. Assistance or treatment should not be delayed until someone has achieved abstinence.

OST, opioid substitution therapy.

† , In 2010 The Lancet published a list of 12 myths about people who inject drugs and HIV, to address misconceptions and stigma held by clinicians.^124^

‡ , The word ‘addiction’ is problematic when applied exclusively to drugs. The state of mind that leads to ‘addiction’ can be applied to relationships and behaviour.

## Appendix 2: Assessing patients during first and subsequent encounters

**TABLE 1-A2:** Examples of scenarios where drug use screening and management is required.

Context	Example
Consulting rooms	Patient requests support for their use of codeine Patient presents with an abscess in the cubital fossa Patient’s parent requests a drug test on their adolescent child Patient requests repeat scripts for tramadol because of non-specific chronic pain
Emergency unit	Patient presents unconscious because of a suspected opioid overdose Patient presents with a suspected stimulant-induced psychosis Patient presents for management of acute wound and found to occasionally inject drugs Male patient presents with recurrent anal discharge and is HIV infected
Hospital wards	Patient is admitted for a primary condition and goes into opioid withdrawal Patient is admitted for infective endocarditis Patient is admitted with TB and found to be HIV/HCV co-infected
Community settings	Community organisation requests doctor to speak about drugs at a community event

HCV, hepatitis C virus, TB, tuberculosis.

**TABLE 2-A2:** The first encounter – The five As of intervention (Ask, Advise, Assess, Assist, Arrange).^129^

Intervention	Description
Ask (history-taking)	mhGAP provides guidance for identifying suspicion of harmful substance use The aim is to identify and document the harmful substance/alcohol use status for each patient at every visit Identify concerns around use Incidental findings indicating harmful substance use, such as signs of drug injecting (‘track marks’) The simplest checklist to start with for adults is the CAGE questionnaire^130^; for youth use TWEAK^131^ CAGE questions:^130^ Have you ever felt you should cut down on your drinking? Have people annoyed you by criticising your drinking? Have you ever felt bad or guilty about your drinking? Have you ever had a drink first thing in the morning to steady your nerves or to get rid of a hangover (eye-opener)? TWEAK is an acronym for Tolerance (T1 number of drinks to feel high; T2, number of drinks one can hold), Worry about drinking, Eye-opener (morning drinking), Amnesia (blackouts), and Cut down on drinking (K/C)
Assess	Conduct a substance use assessment – for the purposes of this guideline we recommend making use of the ASSIST screening tool to stratify risk of harmful substance use, and the ASSIST-linked brief intervention alluded to previously^132^ Other tools which may be used include: AUDIT and DUDIT Determine risk level as per screening tool cut-offs, where broad principles of intervention are as follows: ■ *Low:* feedback, psychoeducation, reinforce non-harmful use, offer continuing support ■ *Moderate:* brief intervention (feedback, advise, assist), consider referral, offer continuing support ■ *High risk:* brief intervention (feedback, advise, assist), arrange referral, offer continuing support Assess for complications or dependence as per the national Adult Primary Care guidelines^133^ Assess for co-occurring mental health conditions using a validated tool. For the purposes of this guideline, we recommend the SAMIS.^134^ Other tools are summarised in Appendix 4. Assess for drug–drug interactions: ■ It is good clinical practice to check drug–drug interactions whenever adding or removing medication, even for a short course. Data on drug interactions is constantly updated. The Liverpool drug interaction site (https://www.hiv-druginteractions.org/) is an excellent resource for determining interactions with ART, is current, and has a strong focus on recreational substance use. For general drug–drug interactions, see https://reference.medscape.com/drug-interactionchecker Diagnostics and work-up (based on clinical history) Drug screening tests (confirmation of opioid use recommended before starting OST) Readiness to change substance-use patterns: ■ *Unwilling to change harmful behaviour*: Provide psychoeducation and offer recommendations offer available assistance, including psychotherapeutic and/or social such as support groups ■ *Ready to modify behaviour*: Provide psychotherapeutic support using techniques such as MI, CBT or CM selected in close consultation with/as is agreed to by the patient
Assist	In all patients, offer brief intervention: for example, psychoeducation plus combination MI- and PST-based counselling where applicable^135^ In patients who are identified as being willing and motivated to modify their harmful/risky substance use behaviour, in addition to brief intervention, engage in treatment Examination Management, linked to outcome of assessment, for example, pharmacotherapy
Arrange	Referral More advanced clinical assessment and care where indicated Provide linkage to other (community-based) health services as per individual patient needs Psychosocial/other services needed Referral to community-based substance treatment programme where the patient desires complete abstinence as an outcome of treatment

ART, antiretroviral therapy; ASSIST, Alcohol, Smoking and Substance Involvement Screening Test; CBT, cognitive behavioural therapy; CM, contingency management; AUDIT, Alcohol Use Disorders Identification Test; DUDIT, Drug-Use Disorders Identification Test; mhGAP, WHO Mental Health Gap Action Programme; MI, motivational interviewing; SAMIS, Substance Abuse and Mental Illness Symptom screener; PST, problem-solving therapy; OST, opioid substitution therapy; CAGE, cut down annoyed, guity, eye-opener.

**TABLE 3-A2:** Follow-up or subsequent encounters.^129^

Risk level	Targeted recommendations
High	Determine whether the patient followed through with the referral Offer additional brief intervention for patients who did not attend the referral Make additional referrals for patients who missed referral Obtain records of assessment and/or treatment for patients who attended referral and/or treatment Discuss ways to help support recommendations of referral source
Moderate	Determine whether the patient reduced or abstained from use For patients who did not make progress with change efforts, acknowledge change is hard Repeat brief intervention and discuss additional ways to support the patient’s efforts For patients who have made changes, reinforce efforts and encourage additional goal-setting Follow-up at subsequent visits
Lower	If the patient indicated that she or he wanted to make a change, ask what, if anything, the patient decided to do about substance use Encourage abstinence from tobacco and illicit drugs and advise low-risk alcohol users to remain within acceptable drinking levels On evidence of escalation of use, conduct brief intervention.

NIDA, National Institute on Drug Abuse.

## Appendix 3: Guidelines for opioid substitution therapy

**TABLE 1-A3:** Taking clinical history for opioid substitution therapy.^74,83^†

Variable	Description
1. History of substance use and treatment	History of substance use (medical indication, recreational exposure, etc.) Methods (e.g. inhaling, occasional injecting, etc.) and patterns of use of this and other substances; quantity of use and consequences thereof To enable comparisons over time and standardisation, a validated screening tool should be used Specifically ask about other medications Source of substances, being mindful of confidentiality and potential disclosure issues Where and when substances are typically taken Usage of substances in situations where danger is posed to them or others, driving and operating machinery, and so on, again mindful of the ethical disclosure issues this may raise Any encounters with law enforcement Previous biomedical interventions around substance use (e.g. detoxification, OST, naltrexone, benzodiazepines, other medication for withdrawal) If history of OST is reported, enquire about: medication, frequency and dosage, duration of treatment and reflections of experience Previous psychosocial interventions (e.g. abstinence-based rehabilitation programmes, mutual or self-help groups)
2. Clinical consequences of use and relevant risk factor assessment	Accidents under the influence Overdose General – loss of weight Cardiovascular – deep venous thrombosis, hypertension, phlebitis, endocarditis, other cardiac complications. If considering methadone, ask about: history of structural heart disease, arrhythmias, unexplained syncope, or medications/drugs that can prolong the QTc interval Respiratory – aspiration Gastro-intestinal – pancreatitis, recurrent gastritis, gastrointestinal bleeds Nervous system – peripheral neuropathy, any central nervous conditions Infectious – TB, HIV, hepatitis, and other blood-borne infections: testing history; diagnosis and treatment Consequences of injecting – vein damage, wounds, abscesses, overdose, etc.
3. Any mental-health-specific diagnoses and interventions	Depression, anxiety and other common mental health conditions Suicide
4. Withdrawal symptoms	Refer to Table 2-A3
5. Social circumstances and support structures	Current living situation (homeless, shelter, with family, etc.) Family, friend and clinical social support, both historic and current Current occupational/school/social structure (community, church, etc.) participation and social functioning.

OST, opioid substitution therapy; TB, tuberculosis; QTc, corrected QT interval.

† , If possible, and with consent, try to obtain collateral history, including from other clinicians.

**TABLE 2-A3:** Opioid withdrawal symptoms.^136^

Early symptoms	Late symptoms
Occurs within 6–12 hours for short-acting and within 30 hours for longer-acting opiates. Symptoms include:	Peaks within 72 h hours and usually lasts a week. Symptoms include:
• Tearing up and nose running	• Nausea and vomiting
• Muscle aches	• Diarrhoea
• Agitation and anxiety	• Goosebumps
• Trouble falling and staying asleep	• Stomach cramps
• Excessive yawning	• Depression
• Sweats	• Drug cravings
• Racing heart and hypertension	
• Fever	

**TABLE 3-A3:** Clinical assessment – differs, based on whether or not the patient is acutely ill.^81^

Non-acute patients	Acutely ill patients
Clinical assessment is routine, with attention to the following: Any signs of hepatic, cardiac or neurological disease; any signs of malnutrition Needle site marks/scars or complications, including groin and neck TB symptom screen and sputum sampling – because of the high burden of TB in South Africa and likely under-diagnosis amongst people who use drugs, take sputum for Xpert MTB/RIF for diagnostic test Note: Opioid use may mask the symptoms of TB as opioids suppress the cough reflex; weight loss is common amongst people who use drugs; opioid withdrawal hot/cold flushes and fever may be like night sweats HIV testing should be offered for those with unknown HIV status or those with HIV-negative status who tested more than 6 weeks earlier Viral hepatitis testing: baseline HBsAg testing should be done, followed by vaccination for those who are not immune; HCV testing should be offered to all people with a history of injecting a substance or with other risk factors† Baseline ECG is recommended for patients with signs or symptoms suggestive of cardiac disease	Standard clinical assessment (alongside) is recommended, with attention to the reason for presentation, in addition to that listed. Clinical assessment: ■ Assess pupil reactions, basic signs (respiratory rate, heart rate, blood pressure, capillary refill). ■ Overdose: sleepiness, dilated pupils, sweating, hypotension ■ Withdrawal reactions, including restlessness, sweating, excessive lacrimation, gooseflesh, dilated pupils, muscle tenderness, increased heart rate and blood pressure ■ See the clinical opiate withdrawal scale: https://www.mdcalc.com/cows-score-opiate-withdrawal#use-cases for an online tool ■ The OOWS has locally been found to have better inter-rater reliability: http://www.emcdda.europa.eu/attachements.cfm/att_35654_EN_OOWS.pdf Laboratory assessment (all performed with relevant counselling and informed consent): ■ TB, HIV, HBV, HCV, syphilis; other infectious diseases as required ■ Monitoring tests in infected patients as per national guidelines ■ In patients with risk factors for liver or renal disease (e.g. chronic alcohol use, chronic hepatitis infection), concomitant medication with risk of hepatotoxicity/nephrotoxicity (e.g. TB and HIV treatment) or signs and symptoms of liver or renal disease (e.g. jaundice, pedal oedema, ascites): LFTs, with INR if evidence of dysfunction; FBC; creatinine ■ Clinically directed testing as indicated

ECG, electrocardiogram; FBC, full blood count; HBsAg, hepatitis B surface antigen; HBV, hepatitis B virus; HCV, hepatitis C virus; INR, international normalised ratio; LFTs, liver function tests; MTB/RIF *Mycobacterium tuberculosis*/rifampicin; TB, tuberculosis; OOWS, objective opioid withdrawal scale.

† , Individuals at higher risk for HCV, as per *South African National Guidelines for the Management of Viral Hepatitis* (2019)^137^ include: people who inject drugs; recipients of blood, blood products and solid organ transplant before 1992; unsafe medical injection practices; occupational exposure; chronic haemodialysis; high-risk/traumatic sexual practices; men who have sex with men; use of intranasal cocaine; piercing, tattooing or acupuncture; surgical procedures without proper sterilisation procedures, traditional/cultural practices such as circumcision and scarification rituals.

**TABLE 4-A3:** Comparison between methadone and buprenorphine (± naloxone) and clinical considerations.^74,81^

Medication	Methadone	Buprenorphine (± naloxone)
Dosing approach	‘Start slow, go slow and aim high’	‘Start low, go fast and aim high’
Pharmacology	Full opioid agonist 24–36-hour half-life	Partial agonist 36–48-hour half-life
Formulation	Oral liquid (2 mg/mL strength)†	Sub-lingual tablet
Dosing	Usually once daily, may need to be taken twice daily in fast metabolisers	Usually daily dosing; can be taken every second day in some cases, particularly during down-titration
Initiation	10 mg – 30 mg initially Patients should ideally be observed for 3–4 hours after the first dose for signs of toxicity or withdrawal. If the patient experiences persistent withdrawal symptoms, a supplementary dose of 5 mg may be considered in a supervised environment. The maximum dose for the first day should not exceed 40 mg.	2 mg – 4 mg initially, with additional doses for symptom control on day 1 (reaching up to 8 mg and sometimes higher) Patients need to be in early stages of withdrawal to avoid precipitated withdrawal; there could therefore be some levels of initial discomfort.
Up titration	Doses need to be gradually up titrated, which may leave the patient with a feeling of unease, causing them to crave and want to ‘top up’. This needs to be managed and discouraged as peak plasma levels are usually only reached after 5 days of treatment. Start low and up-titrate. The risk of overdose is elevated in the first 2 weeks, as the person develops tolerance to methadone. If possible, review the patient daily to titrate the dose against withdrawal symptoms, until the person is clinically stable. If this is not possible, then the person should be seen every 2–3 days and the dose increased only then. Note: As a result of the long half-life of methadone, and accumulation, it is safer for a patient to use small amounts of short-acting heroin than to start with a methadone dose that is too high or is increased too soon. Most people continue to use other opioids for several weeks before reducing/stopping use.	Increase dose by 2 mg – 4 mg daily to a dose that provides stable effects for 24 hour; down-titrate if needed.
Maintenance	Continue to see patient at least weekly and gradually increase by 5 mg/week until cravings stop and withdrawal symptoms are eliminated. For people who inject opioids/have high tolerance, aim for 60 mg/day – 120 mg/day. Some patients require higher doses and others with lower tolerance (e.g. codeine use disorders) stabilise on lower doses. Aim for a dose where the patient feels well, with no/minimal withdrawal, cravings or side-effects and is not over-sedated. Recommended direct observed therapy. Consider take-home dosing when clinically stable with sufficient social support. Ensure medication is locked at home to prevent poisoning by someone else, such as a child. ECG monitoring (to assess QTc intervals‡) is suggested pre-treatment, 1 month on treatment and annually, when doses are > 100 mg or with unexplained syncope or palpitations.	Aim for at least 8 mg/day, to a limit of 32 mg/day, noting that some people will stabilise on lower doses. The correct dose is the one where the patient feels well, with no withdrawal and no or little cravings and no side-effects. Take-home doses are usually initiated earlier Dosing is easier to monitor because of pill format.
Down-titration	Discuss the process with the patient and work with the patient to set goals. Develop a plan for a slow-down titration process that the patient feels most comfortable with, over 3–6 months, reducing by 5 mg/week – 10 mg/week to a level of 40 mg and then by 5 mg/week. Once at a dose of 10 mg – 15 mg, reductions may be 1 mg – 2 mg at a time. The tapering goal should be adapted to each situation and re-evaluated regularly. The dose should be increased if the patient becomes unstable, increases their use of drugs or related harms.	Discuss process with the patient and work with the patient to set goals. Doses > 16 mg daily: may be down-titrated to 4 mg per week or fortnight. Doses 8 mg – 16 mg daily: may be down-titrated to 2 mg – 4 mg per week or fortnight. Doses < 8 mg daily: reduce by 2 mg per week or fortnight. Slower down-titration may be needed.
Missed doses	During initiation phase: ■ ≥ 2 doses, titration should start from initial dose for at least 3 days. During maintenance: ■ ≤ 2 days: usual dose, providing they are not intoxicated. ■ 3 consecutive days: re-evaluate; prescribe 50% of usual dose and increase by 10 mg/2–3 days. ■ ≥ 4 days: restart at 30 mg or less and increase every 2–3 days by up to 10 mg/2–3 days.	During maintenance: ■ If on ≥8 mg and misses ≥4 days, then recommence on 8 mg or half the regular daily dose, whichever is higher.
Pregnant women	First choice for treatment with strong supporting evidence. Opioid-dependent pregnant women should not be required to reduce or stop OST in pregnancy, especially in the third trimester where even mild withdrawal is associated with foetal distress and stillbirth. Main objective is to achieve and maintain stability for the duration of pregnancy and after. If dose reduction is requested, then this should occur in the second trimester, with small frequent reductions, for example, 2 mg – 3 mg methadone every 3–5 days, if there is no illicit drug use and no evidence of maternal withdrawal symptoms. Dose may have to be increased in the third trimester because of increased metabolism. If the patient is sedated after the dose, but experiences withdrawal before the next dose, then split the dose. Next dose, immediately after delivery should be the dose the person was on before the third trimester increases, otherwise they may become overmedicated. Other issues to consider include pain management during and after birth, liaison with obstetricians, paediatricians, childcare during follow-up and other psychosocial interventions.	Increasing evidence base supports use during pregnancy Newer data show no problem of exposure of infant to buprenorphine-naloxone.^137^
Breastfeeding women^137^	Minimal levels of methadone are found in breast milk, regardless of dose. Breastfeeding should be encouraged in women who are stable on methadone OST unless there are other medical contraindications. Breastfeeding may attenuate the severity of neonatal abstinence syndrome and lead to earlier hospital discharge. Contraindications to breast feeding include ongoing illicit drug use.	Buprenorphine is also transferred into breastmilk at low levels, but absorption is expected to be minimal. Limited data on the development of breastfed babies suggest it is safe to use, but a risk-benefit analysis and fully informed maternal consent are recommended.
Risk of overdose	Risk is highest in the first 2 weeks of OST. Because it is a full agonist, there is still a risk of overdose when opioids or other respiratory suppressants are used in conjunction with methadone.^74^ Avoid the use of benzodiazepines, which increase the risk of overdose, and do not reduce the need for methadone.	Reduced risk of overdose because of high binding affinity and partial agonist properties. Risk does still exist, particularly when the effects of the buprenorphine wear off, and when used IV with other sedating substances.
Other risks	Methadone can lead to prolonged QTc interval in some patients. An ECG is usually recommended prior to initiation.	Cytolytic hepatitis and hepatitis with jaundice reported in some individuals. Liver function may need to be monitored.
Diversion	Diversion does occur.	Diversion does occur.
Illicit opioid use	Tends to be higher.	Tends to be lower.
Treatment protocol	Internationally methadone treatment tends to be clinic based initially with directly observed therapy.	In the USA, buprenorphine tends to be more office-based with take-home doses.
Drug interactions	Bioavailability affected by other medications. Doses may need to be altered when patients are on TB, HIV and psychiatric medications. Not to be used with benzodiazepines, alcohol or other sedating drugs. Drug interactions (inhibitors of CYP 2B6 and 3A4 P450 enzymes). Azoles and ciprofloxacin may inhibit metabolism and precipitate toxicity.	Typically, doses need to be increased when patients are on TB and HIV treatment. Drug interactions are rarely clinically significant (inhibitors of CYP 2B6 and 3A4 P450 enzymes).
	For HIV interactions use: https://www.hiv-druginteractions.org/interactions/66944	For HIV interactions use: https://www.hiv-druginteractions.org/interactions/66944
	For HCV interactions use: https://www.hep-druginteractions.org/checker	For HCV interactions use: https://www.hep-druginteractions.org/checker
Costs	Raw material of methadone is cheap and simple to prepare into medication. In South Africa, costs are currently very expensive.	Internationally, buprenorphine is usually more expensive. In South Africa, costs are currently like methadone.

ECG, electrocardiogram; QTc, corrected QT interval; OST, opioid substitution therapy; TB, tuberculosis; HCV, hepatitis C virus; IV, intravenous.

† , This is the formulation of Equity methadone currently available in South Africa. Other formulations (e.g. tablets or power) and strengths exits.

‡ , QTc prolongation is often because of other substances that can also prolong the QTc or inhibitors of the Cyp3A4, usually antidepressants or stimulant drugs.

### Dosing

Optimal dosing is important for full benefits of opioid substitution therapy (OST) to be realised. Opioid substitution therapy should be used for as long as a patient requires it (at minimum, 1 year). The risk of other opioid use is decreased with longer duration of OST.

- Supervised dosing
  ■ It is recommended initially for patients starting on methadone or buprenorphine. Continuation of supervised dosing should be assessed once the patient has been on a stable maintenance dose for approximately 3–6 months and should be individualised.
  ■ Daily dosing may need to continue for patients with limited support structures or for those living in areas where safe storage and access are limited.
- Take-home dosing
  ■ Allowing take-home doses is an important component of patient autonomy and ease-of-use, which enhances retention, the strongest determinant of positive outcomes.
  ■ Take-home dosing allows for patients to focus on other areas of their life because they do not have to spend excessive time and resources to access daily dosing.
  ■ Take-home dosing can initially start over weekends, moving to longer periods of time.
  ■ Discussions with patients and their support network should include available options.
  ■ Community pharmacies are an option for dosing outside of health facilities.
  ■ Buprenorphine (± naloxone) take-home dosing is easier (if in tablet form) and is generally safer than methadone as it has a lower overdose risk.
  ■ Opioid substitution therapy projects that have used methadone report few overdose-related deaths and limited diversion.^80^
  ■ Facilitate a discussion of processes that will be taken if diversion/selling of OST medications becomes apparent, including taking a restorative justice approach and maximising patient safety.
  ■ Supervised dosing should be reinstituted if the clinician has safety concerns or concerns around diversion (e.g. missed appointments, intoxicated while attending appointments, changes in clinical or social situation).

### Regular monitoring of patients

- Once on a stable dose
  ■ Monthly assessment by a doctor, re-prescribing methadone/buprenorphine^75^
  ■ Quarterly assessment of medical and social history
  ■ Repeat assessment of substance use history, using the same tools used at screening (in a non-judgemental manner) – providing counselling and support in relation to outcomes
  ■ Offer HIV and hepatitis C virus (HCV) testing quarterly; assess antiretroviral therapy (ART) adherence as needed
  ■ Perform regular tuberculosis (TB) screening (assess weight loss, cough, fever and night sweats)
  ■ Patients who report ongoing injecting, or injecting in the previous year, should have regular HCV testing.

### Retention and support for adherence

Psychosocial interventions aim to support retention within OST programmes. A broad range of interventions are available and include:

- Social support (which includes addressing basic needs)
- Psychological interventions
- Unstructured supportive therapy (e.g. motivational interviewing [MI])
- Structured interventions (e.g. contingency management [CM] and cognitive behavioural therapy [CBT])
- Group therapy

### Managed opioid withdrawal (detoxification).^74,81^

- The most effective treatment for opioid use disorder is opioid substitution therapy as a long-term management approach. (The South African Standard Treatment Guideline and Essential Medicine List [Adult, Hospital]^136^ guidelines for the medical management of opiate withdrawal [detoxification] are available at: http://www.health.gov.za/index.php/component/phocadownload/category/286-hospital-level-adults).
- Short-term detoxification (a process that requires use of medications over several days per weeks, followed by an expectation of abstinence) is ineffective, yet it is still widely used. It has high rates of recurrent opioid use (up to 90% within 1 year), along with a likely sense of failure or shame in abstinence-focused contexts. Assisted opioid withdrawal is often done as an in-patient procedure. Performing withdrawal management in < 30 days is not recommended.^75^ Withdrawal management should be imbedded within/linked to intensive rehabilitation services to minimise harms should the patient return to use. Ideally, the option to switch a patient to OST should be available if the patient is not able to achieve a goal of abstinence.
- The selection of a substitute opioid is a clinical decision that is made together with the patient after due consideration of: prior response; medical or mental health comorbidities; possible drug interactions; side-effect profile; cost/accessibility; use of other drugs and patient choice.
- Preparation of the patient for withdrawal symptoms is important, and they should be motivated to start a treatment plan, with careful explanations of what they may experience. Withdrawal carries little medical risk but can be very unpleasant. Nevertheless, most initiation can be safely done on an outpatient basis. Principles include gradual decreases in the effects of opiates, either through dose tapering and/or the use of agonists.
- Mild withdrawal reactions can be managed symptomatically, with antidiarrhoeal, antinausea, anxiolytic and analgesic medication. Whilst clonidine can be used for its rapid adrenergic agonist effects on symptoms, the evidence supporting this regimen is limited. Gradual down-titration of the relevant opioid or use of a less potent opioid, can be attempted in more severe situations.
- Benzodiazepines have been associated with fatal overdoses in people with opioid dependency, and their use in the management of withdrawal is discouraged. Risks related to return to the use of opioids after detoxification, particularly amongst people with a history of injecting, include overdose as well as blood-borne infections.

### Management of acute pain in opioid use disorders

A careful history, physical examination and relevant diagnostic studies to identify the cause of the acute pain are the essential first steps.

**TABLE 5-A3:** Assessment and management of acute pain in opioid use disorders.^82,138^

Patients receiving OST	Patients who are actively using opioids
Often require high opioid doses because of tolerance. Patients on methadone maintenance therapy with acute pain should be treated for pain with opioid or non-opioid medications as would be appropriate if they were not on methadone.	The setting of acute pain is not the time to attempt detoxification. Opioid users face stigma and discrimination and may not readily disclose their opioid use. Use a non-judgemental screening tool to assess substance use. Baseline quantity of opioids being used may be difficult to ascertain.
Steps: Confirm the patient’s outpatient daily OST dose and continue this dose. Use multimodal analgesia, in appropriate combinations of short-acting opioid (as required), local anaesthesia and adjuvant anti-inflammatory analgesics and paracetamol. Morphine (short-acting opioid) can be used safely. Doses are higher than in opioid-naïve patients and rapid titration may be needed. Short-acting opioid analgesics should be given on a schedule (every 3–4 hours). On discharge: Provide last methadone dose verification letter, clear instructions for pain management and opioid taper (for pain). Encourage follow-up.	Steps: Plan for inpatient opioid withdrawal management and initiate OST with patient consent, as outlined earlier. Be prepared to titrate opioid doses rapidly if initial doses are ineffective because of tolerance. Arrange outpatient follow-up for OST treatment and pain management.

OST, opioid substitution therapy.

## Appendix 4: Psychosocial and mental health interventions

**TABLE 1-A4:** Suggested screening tools.

	Substance use	Mental health
Adults	ASSIST AUDIT DUDIT CRAFFT S2BI	Substance Abuse and Mental Illness Symptom Screener (SAMIS) Self-Report Questionnaire (SRQ-20) Brief Mental Health Screening Tool (BMH) Kessler 10 Patient Health Questionnaire-9 (PHQ-9) International HIV Dementia Scale (IHDS) Suicide Risk Screening Scale Suicide Risk Screening Tool
Adolescents	ASSIST-Y (10–14 years) ASSIST-Y (15–17 years)	-

AUDIT, Alcohol Use Disorders Identification Test; ASSIST, Alcohol, Smoking and Substance Involvement Screening Test; ASSIST-Y, ASSIST youth; BMH, Brief Mental Health screening tool; CRAFFT, Car, Relax Alone, Forget, Friends, Trouble, substance-related screening tool for adolescents; DUDIT, Drug-Use Disorders Identification Test; IHDS, international HIV Dementia Scale; PHQ-9, Patient Health Questionnaire-9; S2BI, Screening to Brief Intervention; SAMIS, Substance Abuse and Mental Illness Symptom screener, SRQ-20, Self-Report Questionnaire 20.

**TABLE 2-A4:** Summary of brief intervention methodologies.

Intervention	Motivational interviewing	Contingency management	Problem-solving therapy	Cognitive behavioural therapy
Definition	MI is a behavioural intervention designed to help build the patient’s intrinsic motivation to change, as the guiding philosophy underlying the brief intervention component of SBIRT.	CM is an intervention which uses stimulus control and positive reinforcement immediately to change behaviour.	PST is a brief psychosocial intervention to assist individuals in aid of resolving their problems in a step by step format.	CBT is a psychosocial intervention challenging thinking patterns and amending maladaptive behavioural responses.
Indication	Linked to the SBIRT model	Appropriate for those identified as high risk of a substance use disorder or with an active disorder.	Increase in symptoms and ineffective problem-solving skills.	Symptoms that interfere with patient functioning; maladaptive behaviour or thought patterns.
Key principles	See below	Behavioural reinforcement	See below. 4–12 sessions of brief treatment	-

CBT, cognitive behavioural therapy; CM, contingency management; MI, motivational interviewing; PST, problem-solving therapy; SBIRT, screening, brief intervention and referral to treatment.

Resources: https://attcnetwork.org/centers/south-africa-hiv-attc/home

Free Online Tour of MI: https://healtheknowledge.org/course/index.php?categoryid=67

Motivational interviewing (MI) is a psychotherapeutic approach that seeks to move an individual away from a state of ambivalence towards finding motivation to make positive decisions and accomplishing established goals. These goals may include a reduction in harmful behaviour patterns such as harmful substance use or ART non-adherence. The approach to MI includes:

- using the ‘spirit’ of MI to engage with the patient: collaboration, evocation, acceptance and compassion
- using these principles when interacting with the patient: expressing empathy, developing discrepancy (i.e. identifying conflicts between perceptions, behaviours, personal goals and values), avoid argumentation, roll with resistance, support self-efficacy
- assessing the patient’s readiness for change: pre-contemplation, contemplation, preparation, action, maintenance and relapse stages of change
- using the ‘OARS’ as a clinical technique: open-ended questions, affirmations, reflections and summaries

Problem-solving therapy (PST) is a cognitive-behavioural intervention geared at improving an individual’s ability to cope with stressful life experiences. The underlying assumption of this approach is that symptoms of psychopathology can often be understood as the negative consequences of ineffective or maladaptive coping.^139^

Cognitive behavioural therapy (CBT) is a widely used psychotherapy approach. The core theoretical premise is that maladaptive ways of thinking and behaving can generate mental and behavioural problems. The use of CBT ranges from substance use, depressive and anxiety disorders to schizophrenia. It represents a large body of related interventions. These elements include a focus on developing ways of recognising maladaptive thinking and behaviours and then building skills for positive coping to alleviate mental distress and problematic behaviours. It incorporates goal-oriented therapy and some form of talk-based therapy.^139^

Contingency management (CM) is an intervention that provides patients with motivational incentives for meeting pre-determined treatment goals such as abstinence, attendance or medication adherence. The approach is based on principles of behavioural reinforcement. The goal of the treatment is to replace the positive reinforcement obtained from using alcohol and other drugs by providing positive reinforcement for productive behavioural change. Behavioural goals should be set over short time periods (typically 1 week or less) and positive reinforcement must be provided consistently and immediately after the goal has been met.^87^

### Harm reduction counselling tips

The principles of drug set and setting^8^ are very useful in reducing the harms people experience from drug use. If someone is unable to change one aspect of their drug use, then they may be able to make changes in other domains.

**TABLE 3-A4:** UNODC Stimulants guidelines counselling tips.^51,91^

Drug	Mindset	Physical ‘set’	Setting
Improve quality of drug Smaller doses Fewer doses per day Change ways of using Change pattern of using Avoid risky drug–drug combinations	Change drug expectation Increase knowledge Use only when mood or circumstances are best Encourage autonomy and conscious choice	Consider: Sleep patterns Diseases Nutrition Mental health	Policy context Level of surveillance Heightened vigilance All of these can alter drug effect. Should use with others with whom they feel safe. Do not use alone
